# Moderated Online Data-Collection for Developmental Research: Methods and Replications

**DOI:** 10.3389/fpsyg.2021.734398

**Published:** 2021-11-03

**Authors:** Aaron Chuey, Mika Asaba, Sophie Bridgers, Brandon Carrillo, Griffin Dietz, Teresa Garcia, Julia A. Leonard, Shari Liu, Megan Merrick, Samaher Radwan, Jessa Stegall, Natalia Velez, Brandon Woo, Yang Wu, Xi J. Zhou, Michael C. Frank, Hyowon Gweon

**Affiliations:** ^1^Department of Psychology, Stanford University, Palo Alto, CA, United States; ^2^Department of Brain and Cognitive Sciences, Massachusetts Institute of Technology, Cambridge, MA, United States; ^3^Department of Psychology, Yale University, New Haven, CT, United States; ^4^Department of Psychological and Brain Sciences, Indiana University, Bloomington, IN, United States; ^5^Department of Psychology, Harvard University, Cambridge, MA, United States

**Keywords:** online research, cognitive development, meta-analysis, replication, moderated data collection

## Abstract

Online data collection methods are expanding the ease and access of developmental research for researchers and participants alike. While its popularity among developmental scientists has soared during the COVID-19 pandemic, its potential goes beyond just a means for safe, socially distanced data collection. In particular, advances in video conferencing software has enabled researchers to engage in face-to-face interactions with participants from nearly any location at any time. Due to the novelty of these methods, however, many researchers still remain uncertain about the differences in available approaches as well as the validity of online methods more broadly. In this article, we aim to address both issues with a focus on moderated (synchronous) data collected using video-conferencing software (e.g., Zoom). First, we review existing approaches for designing and executing moderated online studies with young children. We also present concrete examples of studies that implemented choice and verbal measures (Studies 1 and 2) and looking time (Studies 3 and 4) across both in-person and online moderated data collection methods. Direct comparison of the two methods within each study as well as a meta-analysis of all studies suggest that the results from the two methods are comparable, providing empirical support for the validity of moderated online data collection. Finally, we discuss current limitations of online data collection and possible solutions, as well as its potential to increase the accessibility, diversity, and replicability of developmental science.

## Introduction

Over the past decade, online data collection has transformed the field of psychological science. Commercial crowdsourcing platforms such as Amazon Mechanical Turk have allowed participants to perform experimental tasks remotely from their own computers, making it easier, faster, and cheaper for researchers to collect large samples. The advantages of online methods led to a rapid increase in their popularity; for example, the percentage of online studies published in three prominent social psychology journals rose from around 3% in 2005 to around 50% in 2015 (Anderson et al., 2019).

Although online methods have been mostly constrained to studies with adults, some recent efforts have pioneered ways to conduct developmental research online (e.g., Lookit, Scott and Schulz, 2017; TheChildLab.com, Sheskin and Keil, 2018; Panda, Rhodes et al., 2020). As the COVID-19 pandemic spurred many developmental researchers to consider safer alternatives to in-person interactions, these methods have quickly gained traction as an innovative way to enable large-scale data collection from children and maximize access and impact in developmental science (Sheskin et al., 2020). Due to the novelty of these methods, however, there is little shared information available about recommended practices for designing, implementing, and executing online experiments with children. Furthermore, researchers may feel hesitant to replicate or build on prior work using online methods because of uncertainties about how developmental data collected online would compare to data collected in person.

The current paper aims to serve as a guide for developmental researchers seeking information about online data collection, with a focus on using video-chat software for moderated (synchronous) data collection. We begin by explaining how moderated methods differ from unmoderated (asynchronous) methods, including their relative advantages and disadvantages. Next, we describe recommended practices and approaches for designing online developmental studies conducted via moderated sessions. In particular, we provide guidelines for implementing two broad classes of measures: forced choice for young children and looking time for infants. To examine the validity of moderated online methods, we present four sets of studies conducted both in person and online that utilize these measures as well as a meta-analysis that compares results from both data collection methods across the four sets of studies. Finally, we discuss the limitations and potential of moderated online data collection as a viable research method that will continue to shape developmental psychology.

## Moderated Online Studies: What It Is and Recommended Practices

Online data collection methods can be categorized as moderated (synchronous) or unmoderated (asynchronous). Unlike **unmoderated (asynchronous) data collection** which functions like Amazon Mechanical Turk, **moderated (synchronous) data collection** functions more like in-person testing; participants engage in real-time interactions with researchers on a web-enabled device using video-conferencing software, such as Zoom, Adobe Connect, or Skype.

An advantage of unmoderated data collection is that it is less labor-intensive than moderated data collection. Participants complete a preprogrammed module without directly interacting with researchers; once the study is programmed, there is little effort involved in the actual data collection process on the researchers’ end. Some pioneering efforts have led to innovative platforms for implementing these modules (Lookit, see Scott and Schulz, 2017; see also Panda, Rhodes et al., 2020), and adaptations of three well-established studies on Lookit have found comparable results to their original in-person implementations (Scott et al., 2017). Its advantages, however, come with trade-offs: due to the lack of researcher supervision, unmoderated data collection is limited to behavioral paradigms where real-time monitoring is not necessary. Thus, this method may not be as well suited for studies where live social interactions and joint-attention are central to the hypothesis and experimental design. Furthermore, adapting an in-person study to an unmoderated module usually involves significant alterations in study procedure and format (Scott et al., 2017), creating additional challenges to directly replicating existing findings in some circumstances.

Moderated data collection, by contrast, is comparable to in-person methods in terms of their costs. It requires recruiting and scheduling participants for an appointment, and at least one researcher must be available to host the session and guide participants throughout the study procedure. Yet, this allows moderated sessions to retain the interactive nature of in-person studies that is often critical for developmental research. Experimenters can have face-to-face interactions with parents and children to provide instructions, present stimuli, actively guide children’s attention, ask questions, and record a number of behavioral measures. Although certain paradigms or measures are difficult to implement even with moderated methods (e.g., playing with a physical toy), many existing in-person studies can be translated into an online version with relatively minor changes in procedures.

Early efforts to apply moderated online data-collection to studies with children have produced promising results, albeit with some caveats. For instance, Sheskin and Keil (2018) collected verbal responses from 5- to 12-year-old children in the United States on several basic tasks via video-conferencing software (Adobe Connect). While children showed ceiling-level performance on questions that assessed their understanding of basic physical principles (e.g., gravity) and fair distribution of resources, their performance on false belief scenarios (i.e., the Sally-Anne task adapted from Baron-Cohen et al., 1985) was significantly delayed compared to results from prior work conducted in person. It is possible that younger children found it more difficult to keep track of multiple characters and locations on a completely virtual interaction; the task also relied primarily on verbal prompts without additional support to guide children’s attention (e.g., pointing). However, because this study did not directly compare the results from online and in-person versions of the same task, it is difficult to draw strong conclusions about the cause of the discrepancies or the validity of moderated methods more generally.

More recently, Smith-Flores et al. (2021) reported replications of prior looking time studies with infants (violation of expectation and preferential looking) via a moderated online format. The findings from data collected online were generally comparable to existing results; for instance, infants looked longer at events where an object violated the principle of gravity than events that did not (e.g., Spelke et al., 1992) and were more likely to learn about object properties following such surprising events (Stahl and Feigenson, 2015)^1^. Contrary to classic work on infants’ understanding of physics, however, infants in this study did not show a sensitivity to violations of object solidity. Although infants in these experiments viewed recorded video clips of events very similar to those used in prior in-person studies, the authors note the experience of viewing such videos on screens is quite different from viewing the event in person, and that differences in the visual properties of test stimuli (e.g., limited aspect ratio of participants’ screens) could have contributed to the discrepancy in results. These concerns might apply to any study using online data collection (both moderated and unmoderated) that involves viewing visual stimuli on a screen as opposed to live events.

In sum, existing data suggest that moderated online studies are indeed feasible, but they also highlight two challenges. First, due to the relative novelty of moderated methods, researchers may be unsure about how to implement a study online and what can be done to minimize potential discrepancies between in-person and online versions. Second, the field still lacks a true apples-to-apples comparison between studies conducted online and in-person using stimuli and procedures matched as closely as possible. In particular, given the variety of dependent measures and procedures used in developmental research, it is important to have a number of such comparisons that span across different experimental designs and methods.

The following sections address these challenges by reviewing current approaches to moderated online study design and providing empirical data that replicate in-person findings with moderated online methods. We begin by outlining key considerations for implementing moderated studies, followed by presentation methods and design considerations that promote participant attention and engagement. Then, we provide concrete examples of implementing dependent measures that are frequently used in developmental research: choice and verbal measures (more suitable for children aged 2 and up) and looking time measures (suitable for infants). We also compare results from experiments that were conducted in-person and adapted for online data collection using these suggestions.

## Moderated Online Studies: Implementation and Recommended Practices

Moderated online studies have been implemented using a variety of video-conferencing software, including Zoom, Adobe Connect, and Skype, among others. Each video conferencing software has benefits and drawbacks that make it better suited for certain research endeavors and styles. There are several particularly important dimensions to consider, including accessibility, functionality, and robustness to technical issues (see Table 1).

**TABLE 1:** Factors to consider when choosing software for moderated online data collection.

Accessibility	Software should ideally be easy to obtain and use, especially for participants. In addition to monetary concerns or internet access (Lourenco and Tasimi, 2020), the need for technical skills, time (e.g., for downloading and installing new software), or specific hardware (e.g., Facetime requires Apple OS) can create barriers to participation. Intuitive software also makes online research easier for both experimenters and participants by reducing time spent setting up and troubleshooting sessions. Using software that many people already have and know how to use can alleviate this issue. Note, however, that accessibility is always relative to a particular population at a particular time; software that is suitable for one population may not necessarily be so for others. For example, Zoom became a more accessible option for conducting developmental research in the United States following the COVID-19 pandemic as more families downloaded and used Zoom in their day-to-day lives for work and remote schooling. As trends in software usage change over time for a given population, researchers should continue to adapt their methodologies accordingly.
Functionality	A software’s user interface, customizability, and security features determine how studies are conducted and the extent to which researchers can customize participants’ online experience. Importantly, security standards regarding recording and storage of online sessions vary across institutions and countries; researchers should keep these in mind when assessing the level of security a given software provides. Additionally, while basic video- and screen-sharing as well as text-chat functionalities are common in most software, the details vary in a number of ways, including how users customize what they can view on screen and how recording is implemented (e.g., local vs. cloud storage). More broadly, intuitive design and real-time flexibility often trades off with precise structure and customization options. Some software (e.g., Adobe Connect) allows experimenters to predetermine the layout of participants’ screens before sessions, and others (e.g., Zoom) automatically generate participants’ layouts and allow participants to modify their layout in real time (following instructions from experimenters). While the former type is ideal for experiments that require precise control over what participants view on screen, the latter type of software is more suitable for sessions involving rapid transitions between multiple experiments with different visual layouts.
Robustness	Recurring lag, audio or video problems, and even login errors can slow down or derail an online session. Although technical issues can also occur in person, issues can be more difficult to resolve in remote interactions where experimenters have limited means to understand participants’ issues. Therefore, it is important to test the frequency and duration of technical issues on both experimenters’ and participants’ ends before committing to a particular video-conferencing software. Depending on the software, screen-sharing or streaming large video or audio files can contribute to unwanted lag or delays. Further, their severity can vary depending on connection speed or devices used by both experimenters and participants. For experiments that rely on precise timing of presented stimuli, researchers might consider presentation methods that do not rely on screen-sharing (e.g., hosting video stimuli on servers or other platforms where participants can access directly, such as online video-hosting or slide-presentation services). If there are consistent participant-end issues that impact the fidelity of a study, researchers can also set explicit criteria for participation (e.g., must use a laptop or cannot use a phone signal-based internet connection).

One common way to implement moderated online studies with young children utilizes locally installed slideshow applications on experimenters’ computers (e.g., Microsoft PowerPoint, Keynote). These applications allow researchers to present a wide variety of stimuli, including images, animations, videos, audio, and written language. Implementing studies using these applications creates a linear structure that naturally segments study procedures into manageable components, making it easy for researchers to manipulate the order of presentation and access notes. Alternatively, studies involving videos, such as many infants studies, have been implemented on video-sharing websites such as YouTube, or slides hosted on cloud services.

One key challenge in designing developmental experiments is ensuring that children stay engaged and attentive throughout the task. On the one hand, an advantage of online data collection is that children participate from their familiar home environments, which could improve their comfort and engagement. On the other hand, however, home environments can be more distracting than lab settings, and researchers have little control over them. For studies that require relatively well-controlled environments, researchers could consider sending parents instructions prior to the testing session to help them create ideal testing environments at home. For example, parents could be instructed to keep siblings out of the room during the session. Here we discuss a few additional strategies to maximize children’s engagement during online data collection and to direct their attention to specific stimuli on screen.

### Elicit Regular Responses From Participants

Because online studies can suffer from technical problems as well as distractions in a child’s home environment, researchers should design them to be robust to frequent interruptions. Eliciting regular feedback from children, either casually or by implementing comprehension questions throughout the task, is one useful strategy. While this is also used in person, frequent questions are particularly useful for identifying long periods of lag or technical issues that can otherwise go unnoticed online. Playing a short video at the start of a session and asking participants to report any lag or audio problems is another quick and easy way to assess participant-end technical issues that might not be readily apparent from an experimenter’s perspective. Finally, it is often useful to make parts of a study easy to repeat in case they are compromised by connectivity issues, audio/video problems, or other unexpected difficulties.

### Use Social Cues

**FIGURE 1:**
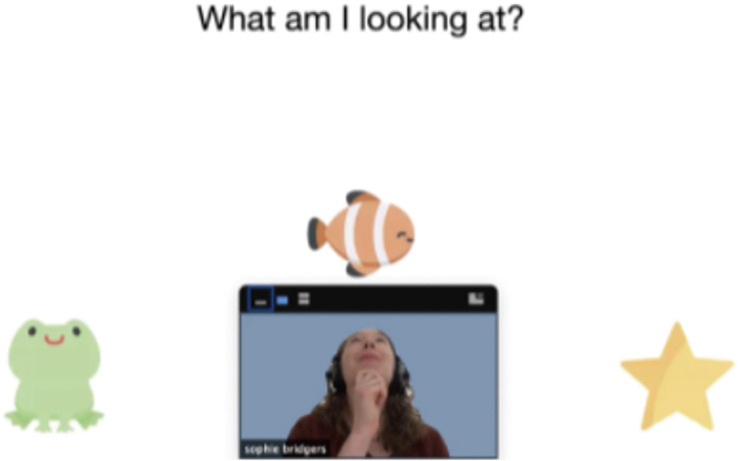
An example screenshot of a moderated session using Zoom. By positioning the experimenter’s video relative to stimuli presented via screen-sharing, the experimenter can use gaze and pointing to elicit joint attention or refer to specific stimuli, similar to how she would use gaze or pointing during in-person interactions. In this example, the participant (or the parent) was instructed to position the experimenter’s video at the bottom of the screen; the experimenter can then “look” at one or more objects on screen and ask the participant to report what she is looking at. Interactions like these can be used as a warmup task to create a “shared reality” between the experimenter and the participant and to facilitate engagement and attention.

In-person studies often utilize social cues from the experimenter (e.g., gaze, pointing) to direct children’s attention. While these are more difficult to use online, some video conferencing software (e.g., Zoom) allows researchers to flexibly adjust the size and location of experimenter’s video feed on participants’ screen, such that the experimenter’s gaze and pointing can be “directed” to specific parts of the stimuli (see Figure 1). These features can be useful for providing the experience of a “shared reality” with the experimenter and can be particularly effective in studies that require joint attention. Additionally, audio and visual attention-getters (e.g., sounds, animations, or markers like bounded boxes that highlight a particular event, character, or object on the screen) can be used instead of experimenters’ gaze or pointing gestures to focus children’s attention on specific stimuli.

### Keep It Short and Simple

Because interacting with others online can tax children’s (and adults’) cognitive resources more than in-person interactions (e.g., Bailenson, 2021), it is important to keep online studies as short and simple as possible. For studies that require relatively longer sessions, presenting them as a series of multiple, distinct activities can help maintain children’s attention and enthusiasm throughout. In cases where concerns about cross-study contamination are minimal, researchers can also run more than one experiment per session. Of course, different studies have different attentional demands and require varying levels of continuous attention. Thus, researchers should consider what counts as a consequential lapse of attention and devise their exclusion criteria accordingly during the pre-registration process.

As we emphasized earlier, one key advantage of moderated methods is the relative ease of adapting in-person studies to an online format without significant changes to the procedure. This means that many of the strategies used to promote attention and engagement in person also apply to online studies. For instance, color-coding and animating stimuli, using engaging stories and characters, and talking in simple, plain language can also help children stay engaged. Overall. relatively minor changes to the way that stimuli are presented can have a large impact on children’s attention and engagement throughout an online session.

In what follows, we provide more specific guidelines for implementing two kinds of dependent measures (choice and looking time) with concrete example studies for each type of measure. Importantly, these studies address different theoretical questions and have not been fully published at the time of writing this article; the key reason for reporting these datasets is to examine the validity of moderated online data collection. As such, we describe the hypotheses and methods of these studies only to the degree necessary to contextualize our analyses: comparing the main effect of interest from data collected in-person versus online. In addition to a direct comparison of their results, we present a meta-analysis of all four sets of experiments that provides further evidence that moderated online and in-person testing yielded similar results across the current studies.

## Examples and Replications I: Choice and Verbal Measures in Moderated Online Studies

**FIGURE 2:**
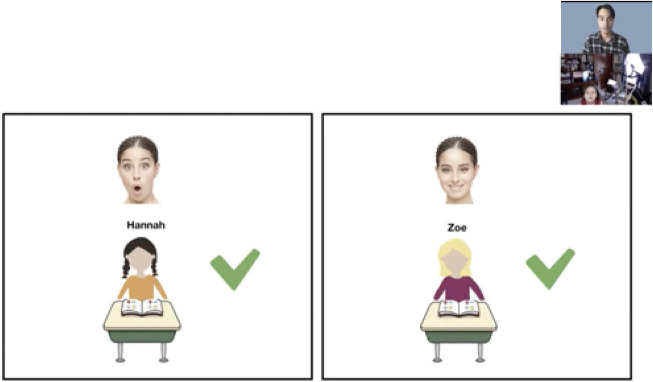
One option for eliciting choice in Study 2. Children could be asked to choose which agent is better at math, Hannah in orange or Zoe in purple?

To elicit explicit choices from children who are old enough to understand verbal instructions, in-person studies often use pointing or reaching as dependent measures. These behaviors, however, can be difficult to assess in online studies; webcam placement can vary across participants, and participants may move outside the field of view during the critical response period. One useful approach for implementing choice tasks for children in this age range is to replace pointing or reaching with verbal responses, and associate each choice with overt visual cues such as color. For example, a binary choice question can be presented as a choice between one character wearing orange and another character wearing purple (color assignment counterbalanced), with children only needing to choose “orange or purple” (see Figure 2). In these choice paradigms, it is important to keep the on-screen location of key choices or stimuli as consistent as possible throughout the study such that transitioning between slides is less disruptive and easier to follow.

In addition to forced-choice measures, experimenters can elicit free-form verbal responses or actions as dependent measures, or ask the parent to type out the child’s responses via text chat. Researchers can also implement other creative dependent measures, such as prompting children to make a drawing and share it with the experimenter via video. As long as a behavior can be consistently prompted and recorded, it can likely be used as a measure in a moderated online study. Here, we present two additional sets of studies conducted online and in person that measured children’s explicit choice between two agents. One study examined 4- to 5-year olds (Study 1) and another examined 6- to 9-year olds (Study 2).

### Study 1

#### Research Question

Can 4- to 5-year-old children use information about task difficulty to infer relative competence when agents’ efforts are matched? To investigate this question, children viewed two agents who used 10 wooden blocks to build different structures; one placed the blocks on top of each other to create a vertical tower while the other placed them next to each other to form a horizontal line. Children were then asked which agent was better at building blocks. Prior work has established that children understand that the vertical structure is “harder” (i.e., takes longer) to build compared to the horizontal structure (Gweon et al., 2017). Thus, the hypothesis was that even though both agents moved and placed 10 blocks, if they took equally long to finish building, children would judge the agent who built the vertical (and therefore harder) tower as more competent than the agent who built the horizontal line.

#### Participants

##### In-person

Twenty 4- and 5-year-old children participated in-person at the Boston Children’s Museum (10 females, mean: 62.25 months, range: 49–71); 10 additional children were tested but excluded due to failing the practice question (*n* = 3) or inclusion criteria question (*n* = 7).

##### Online

Twenty 4- and 5-year-old children participated online (nine females, mean: 59.23 months, range: 49–71). Participants were recruited via local and online advertising. Seven additional children were tested but excluded due to failing the inclusion criteria question (*n* = 3), technical issues (*n* = 1), declining video (*n* = 1), not wanting to answer the inclusion question (*n* = 1) or dropping out (*n* = 1).

#### Methods

##### Both in-person and online

An experimenter first asked children “Who is better at writing letters — you or your parents?” and then “Who is better at playing on the playground — you or your parents?” If children chose themselves for writing or their parents for playing, they were corrected. In the test video, children watched two agents build block structures. Below one agent was a picture of a 10-block vertical tower and below the other agent was a picture of a 10-block horizontal tower. We chose these structures based on findings from Gweon et al. (2017) showing that 4-year olds readily judge the 10-block vertical structure as harder to build than the 10-block horizontal structure based on static pictures of the initial states (i.e., scattered blocks) and final states (finished towers), without seeing the building process. The agents first said they wanted to build a pictured tower. One agent pointed to the picture below her and said, “I’m going to make this,” then the other agent repeated the same action. Next, the agents began to build at the same time. A screen blocked visual access to the agents’ building actions. Both agents indicated they were finished building at the same time. The screen then lifted, revealing what each agent made. Children were then asked the test question followed by an additional question used as a part of the inclusion criteria. Those who answered the inclusion question inaccurately were excluded from analyses.

##### In-person

Before the test trial, children watched a practice video where two agents drew shapes, finishing at different times. While the agents drew, a screen blocked them. One of the agents indicated she was done drawing, followed by the other agent a few seconds later. Then the screen lifted to reveal what they made. Children were asked which agent finished first and whether the agents had made the same or different pictures. If they answered incorrectly, they were excluded from analysis. Afterward, children viewed the test trial, and were subsequently asked the critical test question: “Who is better at building blocks?” and were encouraged to point, followed by the inclusion question “Which tower is better?”

##### Online

The online study was the same as the in-person study except for the following modifications. To make the study amenable to online testing, children’s attention toward desired locations in the presentation was cued using animation and sound. Instead of asking children to point to which agent was better at the end, they were instructed to make their choice based on the color of squares surrounding each agent. To reduce study time, the practice trial was also removed (more than 93% of children passed the practice trial in in-person versions of three similar prior studies). Finally, we changed the inclusion question to “which tower is harder to make?”

#### Results

##### In-person

Children’s performance on the test question was significantly above chance (90%, CI = [80%, 100%], *p* < 0.001). This result held even after including the seven children who failed to answer the inclusion question accurately (74%, CI = [60%, 93%], *p* = 0.02).

##### Online

Consistent with in-person findings, children’s performance on the test question was significantly above chance (85%, CI = [70%, 100%], *p* = 0.003). See Figure 4 (1) for a summary of results.

**FIGURE 3:**
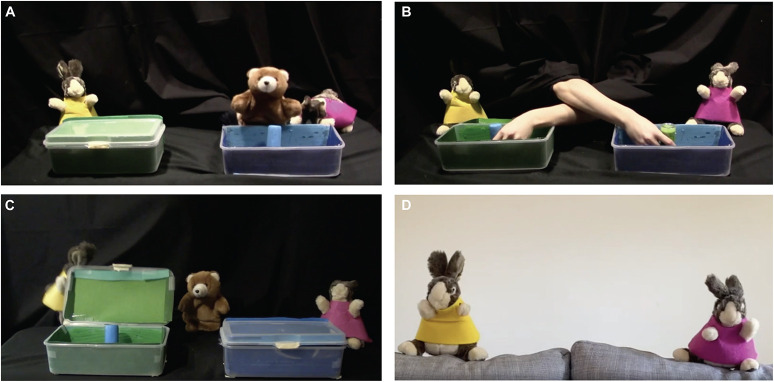
Screenshots of video stimuli implemented in Study 3 (preferential looking, see Woo and Spelke, 2020). **(A)** Participants were first familiarized to the bear’s preferred toy. **(B)** The contents of the boxes were then switched either in the rabbits’ presence or absence. **(C)** One rabbit opened the box where the desired toy was moved to while the other opened the one where the desired toy was originally. **(D)** At test, infants were shown the two rabbits. In person, infants were asked to choose the one they like and their reach was recorded; online, infants were presented with a video of the two rabbits and their preferential looking was measured.

**FIGURE 4:**
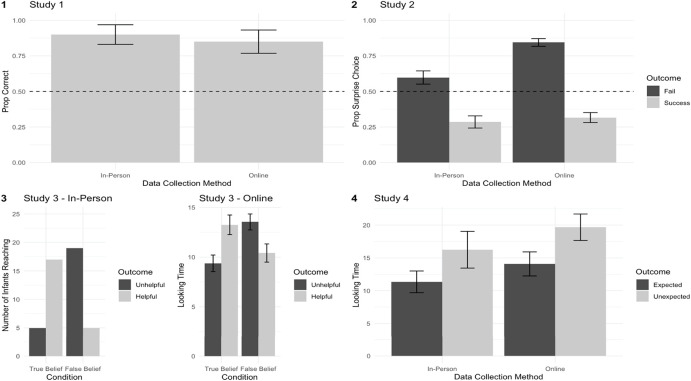
Summary of results across Studies 1–4, comparing in-person and online data collection methods. Error bars indicate standard error.

### Study 2

#### Research Question

Do children use an adult’s expressions of surprise to draw inferences about others’ competence? The in-person data was first reported in a study by Asaba et al. (2020). Children were shown two students who both succeeded or failed at a task (e.g., a math problem), accompanied by their teacher’s reaction; the teacher responded with a surprised expression to one and an unsurprised expression to the other. Children were then asked which student was better at the task. The hypothesis was that children would use the teacher’s surprise to infer the students’ competence; a teacher’s surprise at a student’s failure likely indicates competence whereas the same surprised expression in response to a student’s success indicates a lack of competence.

#### Participants

##### In-person

Twenty-eight 4- to 9-year-old children (mean = 79.2 months, range 49.2–118.8 months; 13 girls, 15 boys) participated in-person at a museum (*n* = 20) and campus preschool (*n* = 8) in Palo Alto, CA, United States. Participants who did not respond to the test questions (i.e., responded “both” to all questions; *n* = 16-year-old) were excluded.

##### Online

Ninety 6- to 8-year-old children (30 6-year olds, 30 7-year olds, 30 8-year olds; mean age = 90 months, range = 72–106.8 months; 48 girls boys, 42 boys girls). Participants were recruited via local and online advertising. An additional child was tested and excluded due to having audio problems during the testing session (pre-registered exclusion).

#### Methods

##### Both in-person and online

Subjects were first introduced to a teacher and her two facial expressions, described as “surprised” and “non-surprised,” respectively. Then, all participants underwent the key trials. In each trial, two students either both succeeded or failed an activity, and the teacher expressed surprise to one student while expressing no surprise to the other (henceforth “surprise student” and “non-surprise student,” respectively). Specifically, the experimenter first remarked on one student’s performance outcome (either a success: “Look, Hannah got the math problem right!” or a failure: “Look, Hannah got the math problem wrong!”), revealed the teacher’s emotional response to the outcome (either a surprised or non-surprised face), and asked a check question: “Is the teacher surprised or not surprised?” If participants provided an incorrect response to the check question, the experimenter corrected them. This sequence was repeated for the other student (e.g., “Zoe”; gender-matched) in the trial who performed exactly the same but received the other emotional response. Finally, with images of the students’ outcomes and the teacher’s expressions visible, the experimenter asked, “One of the kids is better at this game. Who is better?” Children then indicated their response.

##### In-person

Children viewed eight trials, consisting of four different activities (math, spelling, kicking, and throwing) and two types of outcomes (success, failure) for each. After each trial, children indicated their response by pointing or responding verbally with the student’s name.

##### Online

Children only saw four trials instead of eight to reduce the length of the online experiment. The four trials consisted of two activities (randomly selected from the four activities in the in-person study) with two types of outcomes for each. Participants responded by saying the student’s name aloud.

#### Results

##### In-person

As a group, children chose the non-surprise student in success trials (71.4%, *Z* = 2.91, *p* = 0.004, Exact Wilcoxon-Pratt Signed-Rank Test), but did not choose the surprise student in fail trials significantly above chance (59.8%, *Z* = 1.32, *p* = 0.211). Given the wide age range, we median-split children into younger (age: 4.1–5.9; *N* = 14) and older age groups (age: 6.2–9.9; *N* = 14) and looked at children’s choices within each trial. The older group was accurate for both success and fail trials (Success: 98.2%, *Z* = 3.64, *p* < 0.001; Fail: 76.8%, *Z* = 2.16, *p* = 0.039). The younger group was at chance for both trial types (Success: 44.6%, *Z* = −0.58, *p* = 0.71; Fail: 42.9%, *Z* = −0.81, *p* = 0.54) with no difference between success and fail trials (*Z* = 0.06, *p* = 0.99).

##### Online

As a group, participants (age: 6.0–8.9; *N* = 90) chose the non-surprise student in the success trials as more competent (Mean proportion = 68.33%, *Z* = 3.62, *p* < 0.001 (Wilcoxon Signed-Rank Test) and the surprise student in fail trials (Mean proportion = 84.44%, *Z* = 6.85, *p* < 0.001, Wilcoxon Signed-Rank Test). These results are comparable to the results of the older group (age: 6–9) in the in-person study. Note that the online version of the task found an interaction between age and outcome, whereas in-person studies additionally found significant main effects of outcome and age. However, this difference may be due to the fact that online studies had a more limited age range. See Figure 4 (2) for a summary of results.

## Examples and Replications II: Looking Time Measures in Moderated Online Studies

The choice measures described in Section “Examples and Replications I: Choice and Verbal Measures in Moderated Online Studies” are relatively straightforward to adapt online, but they can only be used with children who are old enough to follow verbal instructions. To explore how infants can be studied using moderated online methods, here we discuss ways to implement looking time measures, including preferential looking and violation of expectation (VoE) paradigms (see also Smith-Flores et al., 2021). As in Section “Examples and Replications I: Choice and Verbal Measures in Moderated Online Studies,” we review two sets of studies conducted in person and online implementing these measures. They demonstrate both the feasibility of conducting infant research online and that data collected online can yield comparable results to data collected in person.

Preferential looking is relatively straightforward to implement via moderated methods. It has traditionally been used to measure the preferences of infants who are too young to reach (e.g., Kinzler et al., 2007; Hamlin et al., 2010; Powell and Spelke, 2018); indeed, prior work has shown that younger infants often look at the same characters that older infants ultimately reach for. Preferential looking paradigms can be implemented online by presenting stimuli side by side and assessing the direction and duration of participants’ gaze; Study 3 presents an example implementation (see Figure 3). When using preferential looking as a dependent measure in online studies, infants’ positioning with respect to the camera’s field of view is important; although preferential looking studies can also be implemented using unmoderated methods, in moderated sessions, an experimenter can provide clear, real-time instructions to the parent about how best to position or reposition their child.

Violation of expectation paradigms (Aslin, 2007) can also be implemented online, using either unmoderated or moderated methods. Below we provide an example of a moderated online study (Study 4) where infants were shown sets of video stimuli for familiarization and then a test stimulus presented as a separate video; infants’ duration of looks at the test video was measured in the same way as an in-person VoE paradigm. Note that in online VoE studies, variability in camera angle, screen size, and video feed quality can make it hard for experimenters to determine when infants divert their gaze from the screen and for how long. Therefore, successful implementation of VOE studies in moderated sessions require a reliable method for tracking infants’ gaze and duration in real time. To address this issue in Study 4, two coders individually tracked and measured infants’ looking duration^2^. In general, variability across participants in experimental setup is an important factor to consider when deciding exclusion criteria tailored for online data collection.

In what follows, we present the key methods and results from two sets of studies with infants conducted in person and online. One examines 15-month-old infants’ preferential reaching and looking (Study 3) and another examines 6- to 13-month-old infants’ looking time (VoE, Study 4).

### Study 3

#### Research Question

Do infants’ social evaluations take into account the intentions of agents acting on false beliefs? The methods, analyses, results, and discussion of Study 3 were first reported fully in Woo and Spelke (2020). Infants viewed scenarios with social agents who possessed true or false beliefs about the outcomes of their actions directed toward another agent in need of help. Infants’ preferential reaching (in-person) or preferential looking (online) toward the agents were measured. The primary research question in Woo and Spelke (2020) is whether infants prefer agents with helpful intentions or agents who cause positive outcomes.

#### Participants

##### In-person

Forty-six infants (23 females, mean: 15.06 months, range = 14;10–15;18) participated in person. An additional 15 participants were excluded, based on preregistered exclusion criteria (see Woo and Spelke, 2020, for full details about demographics and about exclusion).

##### Online

Forty-eight infants (26 females, mean: 14.91 months, range = 14;10–15;20) participated online, using Zoom’s screen share features. Participants were recruited via local and online advertising. An additional four participants were excluded, based on preregistered exclusion criteria (see Woo and Spelke, 2020).

#### Method

##### Both in-person and online

Woo and Spelke (2020) familiarized infants to videos of puppet shows in which a bear protagonist repeatedly grasped a toy (its desired toy) in a box while two rabbits observed. Following familiarization, the toy was moved to a new box and both boxes were closed, either as the rabbits were present or absent. In their presence, the rabbits would have true beliefs about the location of the desired toy; in their absence, they would have false beliefs. In the final event, the bear returned. One rabbit opened the original box that had contained the bear’s desired toy, whereas the other rabbit opened the new box that contained the bear’s desired toy.

##### In-person

Infants sat on their caregiver’s lap in the lab before a 102-cm by 132-cm LCD projector screen. After viewing all events, an experimenter assessed infants’ evaluations through their preferential reaching behavior directed at the two rabbits. The experimenter determined the infant’s choice as the first rabbit they touched via a visually guided reach (see Woo and Spelke, 2020, for reliability analyses).

##### Online

The online version of this experiment was almost exactly the same as in-person, except infants’ evaluations were assessed through their preferential looking toward the two rabbits, presented via Zoom screen share. After the final event, the two rabbits appeared on opposite sides of the screen and moved to an experimenter’s pre-recorded voice saying “Hi! Look! Who do you like?” three times, once every 10 s (see Figure 3D); infants’ looking toward each rabbit was then assessed (see Woo and Spelke, 2020, for reliability analyses).

#### Results

##### In-person

Woo and Spelke (2020) found that, when rabbits had true beliefs about the desired toy’s location, infants preferentially reached for the rabbit who opened the new box with that toy (17/22 infants, binomial *p* = 0.016, relative risk = 1.54). By contrast, when rabbits falsely believed that the desired toy was in its original box, infants reached for the rabbit who opened the original box (19/24 infants, binomial *p* = 0.006, relative risk = 1.58). The patterns of reaching based on outcomes (i.e., which box rabbits opened) differed significantly between conditions [χ^2^(1) = 12.47, *p* < 0.001, Wald’s odds ratio = 12.92].

##### Online

When rabbits had true beliefs about the desired toy’s location, infants preferentially looked at the rabbit who opened the new box with that toy [mean preference% = 58.2%, 95% CI [51.9%, 64.5%], SD = 14.9%, one-sample *t*(23) = 2.71, *p* = 0.012, *d* = 0.55]. When rabbits falsely believed that the desired toy was in its original box, infants instead preferentially looked at the rabbit who opened the original box [mean preference% = 57.0%, 95% CI [50.8%, 63.2%], SD = 14.6%, one-sample *t*(23) = 2.36, *p* = 0.026, *d* = 0.48] (Figure 3B). Looking preferences based on outcomes differed significantly between conditions [two-sample *t*(45) = 3.59, *p* < 0.001, *d* = 1.03]. See Figure 4 (3) for a summary of results.

### Study 4

#### Research Question

Do infants expect other agents to minimize the cost of their actions? The in-person version of this study was previously published as Experiment 1 in Liu and Spelke (2017). Infants were shown efficient and inefficient actions after a habituation (in-person) or familiarization (online) period, and their duration of looking toward those actions was measured. The hypothesis was that if infants expect an agent to perform an efficient action, then they will look longer when they perform an alternative, inefficient action^3^.

#### Participants

##### In-person

Twenty 6-month-old infants (10 females, mean age = 5.95 months, range = 5.6–6.3) participated in-lab. Seven additional infants were tested but excluded from the final sample (two did fussiness, one did not habituate, two because of experimental or technical error, and one for interference from caregivers) based on exclusion criteria specified ahead of data collection.

##### Online

The online replication included 27 infants ranging from 6 to 13 months of age (15 females, mean age = 10.4 months, range = 6.9–13.1). Participants were recruited via local and online advertising. Two additional infants were tested but excluded from the sample (one due to fussiness and one for failing to complete the test trial) based on exclusion criteria specified ahead of data collection. The age range for this sample was chosen to match or exceed the age of infants in the original study. The sample size was chosen based on a simulation-based power analysis, implemented using the simr package in R (Green and MacLeod, 2016), over the confirmatory analysis from the original study (comparison of looking time between the inefficient and efficient events).

#### Methods

##### Both in-person and online

Infants were first calibrated to the display screen using a toy that was held at the center, top, bottom, left, and right of the screen (in-person), or a video of an object that appeared at each of those locations (online). Parents were instructed not to engage with their infants or attract their attention toward or away from the stimuli. During the study, infants first saw looped videos of an agent leaping over a tall barrier. The height of this barrier varied slightly across loops, and the agent always conformed the height of its jump to the height of the barrier, following previous studies (Gergely et al., 1995). Then, at test, the barrier was removed and replaced with a lower barrier that infants had not seen before. Infants, on alternating trials, then saw the agent jump the same height as before (now inefficient) or jump low enough to just clear the new barrier (now efficient). The order of test events was counterbalanced across participants. All trials lasted until babies looked for 60s, or until they looked away for two consecutive seconds. The final data generated for the analysis was coded from video recordings by researchers who were naive about the order of test events presented to infants.

##### In-person

Infants sat on their caregivers’ laps in front of a large projector screen. Infants saw 6 to 14 habituation trials, and 3 pairs of test trials. Infants met habituation criteria when their summed attention across the most recent 3 trials fell to below half of their summed attention across the first 3 trials, or after 14 habituation trials. For more details, see Liu and Spelke (2017).

##### Online

Infants viewed stimuli presented as a YouTube playlist on parents’ laptop or tablet screens in their homes. They either sat on their caregivers’ laps or in a high chair. An experimenter used Zoom’s screen share and remote control features to move through the playlist and record the session. To simplify the study design, infants saw only six familiarization events and two pairs of test trials. The experimenter determined trial duration using jHab (Casstevens, 2007) and went to the next video in the playlist when indicated. Because of the variable screen sizes and setups across infants, and the lower quality of the videos from the study sessions, two naive coders generated the final data rather than one; the initial coding took place during the session, and coders also reviewed the recordings afterward for accuracy. Neither had access to the view of the stimuli. If they disagreed about the duration of looking by more than 4s, or about whether a particular trial should be included or excluded, a third coder resolved the disagreement. In two cases, no video record of the session could be recovered, so the original coding generated during the experiment was used.

#### Results

Across both studies, the primary dependent measure was the average looking time toward the unexpected (i.e., inefficient action) vs. expected (i.e., efficient action) test event, log transformed to correct for skew in the data.

##### In-person

Infants looked longer at the familiar but inefficient jump versus the novel but efficient jump (M_ineffcient = 16.25, M_efficient = 11.35, [−0.49, −0.11], β = −0.46, *B* = −0.3, SE = 0.1, *p* = 0.006, two-tailed, mixed effects model with looking time in log seconds as the response variable, trial type as a fixed effect, and a random intercept for each participant, all subjects included based on a 4/n cutoff from Cook’s Distance, where n is the number of participants (Nieuwenhuis et al., 2012).

##### Online

Infants who participated online performed similarly to those that participated in-person: infants looked longer at the inefficient jump versus the efficient jump (M_ineffcient = 19.68, M_efficient = 14.08, [−0.7, −0.14], β = −0.611, *B* = −0.36, SE = 0.13, *p* = 0.012, two-tailed, mixed effects model with looking time in log seconds as the response variable, trial type as a fixed effect, and a random intercept for each participant, removing one influential participant identified using Cook’s Distance). Including this participant did not change the interpretation of the predicted effect. See Figure 4 (4) for a summary of results.

## Evaluating the Validity of Online Developmental Methods: A Meta-Analysis

While the disruption of in-person testing was an unavoidable consequence of the COVID-19 pandemic, it also provided a rare opportunity to directly compare results across two nearly identical versions of studies administered online and in-person. Collectively, the four studies presented in Sections “Examples and Replications I: Choice and Verbal Measures in Moderated Online Studies” and “Examples and Replications II: Looking Time Measures in Moderated Online Studies” successfully replicated the initial findings from in-person procedures using online (moderated) adaptations of the same procedures. In order to facilitate comparison of effect sizes across different studies using different dependent measures, we conducted a meta-analysis based on data from Studies 1–4. Meta-analysis is a standard method for aggregating effect sizes across disparate experimental paradigms (Hedges, 1992), and meta-analyses can even be effective ways to aggregate across small numbers of studies (Goh et al., 2016).

**FIGURE 5:**
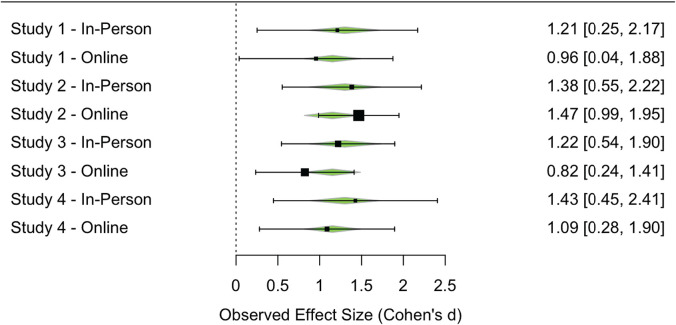
Forest plot showing the standardized effect of interest across Studies 1–4, comparing in-person and online data collection methods. Points are sized based on sample, and error bars indicate effect size variance. Green triangles show random-effects multilevel meta-regression model estimates of the effect size for each study.

For each study reported here, we calculated the effect size and associated variance for the primary effect of interest. We calculated Cohen’s d for Studies 3 (online) and 4 (in-person and online) and log odds for the remaining studies. We then converted all effects to Cohen’s *d* and computed variance using the *compute.es* package in R (Del Re and Del Re, 2012). We then performed a random-effects multilevel meta-regression over the eight effect sizes using *metafor* (Viechtbauer, 2010). This meta-regression attempted to estimate the effect of online data collection, predicting this effect across the four pairs of experiments. A forest plot is shown in Figure 5. In aggregate, the meta-analysis estimated a small negative, non-significant effect of data collection method (online), *p* = 0.58, 95% CI [−0.67, 0.38]. This finding confirms the impression that, despite the differences in context and implementation, data collected in person and online elicited similar effects in the current studies, providing empirical support for the validity of moderated online data collection.

## Discussion

Online developmental studies are still in their infancy, and the idea of using video-conferencing tools for developmental studies may be new to many researchers. To help researchers decide how best to implement their own online studies, here we reviewed various considerations for software choice as well as techniques and strategies for designing effective studies that maximize participant attention and engagement. We then presented four examples of studies where an in-person experiment was replicated by adapting the procedures and stimuli for moderated online data collection.

Comparison between in-person and online studies suggests that moderated online data collection provides a viable alternative to in-person data collection. In Study 1, preschoolers’ choice of agent in person was nearly identical to those who participated online. Similarly, in Study 2, elementary schoolers performance on the test question was significantly above chance in both the in-person and online versions of the study. In Study 3, infants’ pattern of preferential reaching measured in person closely paralleled the pattern of infants’ preferential looking measured online. In Study 4, infants’ looking times across two conditions were comparable between the in-person and online versions of the study. Further, a meta-analysis revealed similar effect sizes across in-person and online data collection for the studies in the current sample.

### Limitations

Although the overall results of the current studies suggest similar experimental outcomes for developmental studies conducted in-person and online, there are several factors that limit the generalizability of these findings and our ability to draw sweeping conclusions about online research as a whole. First, the current studies focus primarily on social cognition and therefore feature animated agents that exhibit various behaviors. The presence of such agents may have made these studies particularly interesting and engaging to infants and children (see Kominsky et al., 2020). Whether similar results would be expected in studies that only involve inanimate objects, shapes, or sounds remains an open question.

Second, the current studies utilize a small subset of possible measures (i.e., verbal choice, preferential looking, and looking time). The efficacy of other, more continuous measures, such as rating scales or free form responses, is less clear. Therefore, future research is needed to examine the viability and efficacy of a broader range of methods, measures, and research questions. Nonetheless, the current data suggest that the results of online developmental studies, when adapted properly, are comparable to those of similar studies conducted in person.

Third, these studies were conducted in the United States with participants who have relatively reliable internet access and are reasonably comfortable operating laptops, tablets, or smartphones. Therefore, the current results do not speak to the efficacy of online research in populations with unreliable internet access or less experience with telecommunications technology. Nonetheless, online data collection, in principle, offers easier access to some samples—particularly those in developing countries with increasing internet-access—than traditional in-person data collection. Thus, we see online methods as a promising approach for improving the diversity, generalizability, and outreach of developmental research, and more broadly as an exciting direction of future research efforts that are larger in scale and impact (Sheskin et al., 2020).

### Future Developments

Obviously, certain kinds of studies simply cannot be adapted to an online format, especially if they require special equipment or interaction with physical stimuli (e.g., neural measures, physical exploration tasks, etc.). Looking back at the past several years, however, many new strategies have been developed to collect various dependent measures using online data collection that were believed to be infeasible. We hope this trend continues, and look forward to new and exciting methods, measures, and research questions that can be implemented online. As online methods become more easily accessible and widely adopted, researchers across a greater variety of subdisciplines will adapt their studies to an online format. In turn, this will bring a greater variety of dependent measures and experimental paradigms. While our work provides preliminary support for some existing approaches, further research is needed to determine their efficacy compared to alternative approaches.

Online research allows researchers to easily expand the size and the demographics of their samples, with the potential to reach families across the world (Sheskin et al., 2020). In addition to the positive impact on the representation and generalizability of developmental research, this provides an unprecedented opportunity to improve community outreach and engagement. Although this can happen passively as more families participate in the scientific process, researchers can also actively update families on research findings and speak directly to those interested in developmental research. Conducting research online also makes it easier for students to get involved in the research process, especially those who may have limited access to universities with traditional in-person developmental research facilities. As resources for online research become more centralized, we hope more individuals take part in the scientific process as both researchers and participants.

## Conclusion

Online developmental studies proliferated in part because of the COVID-19 pandemic, but they are likely here to stay. Here, we have described a number of countermeasures to the limits of the online medium, including ways to minimize the impact of technical issues and adapt developmental stimuli for online use. We also presented a meta-analysis of developmental studies conducted in-person and online, and found comparable results between both versions. Our main goal was to demonstrate the feasibility and promise of conducting developmental research online. With these initial steps, we hope that researchers continue to utilize the medium to innovate and improve the accessibility, diversity, and replicability of developmental science.

## Data Availability Statement

The data for Studies 1, 2, and 4 can be found in the following repository: https://osf.io/rpvxw/. See Woo and Spelke (2020) for Study 3 data.

## Ethics Statement

The studies involving human participants were reviewed and approved by Stanford University IRB, Harvard University IRB, MIT IRB. Written informed consent to participate in this study was provided by the participants’ legal guardian/next of kin. Written informed consent was obtained from the minor(s)’ legal guardian/next of kin for the publication of any potentially identifiable images or data included in this article.

## Author Contributions

AC and HG drafted the initial manuscript. SB, BC, GD, TG, MM, SR, JS, NV, and XZ helped develop online testing materials. MA, BC, JL, SL, BW, and YW contributed the empirical data and drafted the study methods. AC and MF conducted the meta analyses. All authors contributed to the article and approved the submitted version.

## Conflict of Interest

The authors declare that the research was conducted in the absence of any commercial or financial relationships that could be construed as a potential conflict of interest.

## Publisher’s Note

All claims expressed in this article are solely those of the authors and do not necessarily represent those of their affiliated organizations, or those of the publisher, the editors and the reviewers. Any product that may be evaluated in this article, or claim that may be made by its manufacturer, is not guaranteed or endorsed by the publisher.

## Footnotes

1 This study also included a successful replication of Wu et al. (2017) on infants’ understanding of emotional expressions.

2 Another solution is to use an infant-controlled looking procedure in which trial length is determined by an individual infant’s looking behavior, but such procedures can be difficult to implement. For unmoderated studies, the Lookit platform has recently begun to support this feature.

3 Further experiments helped constrain the interpretation that infants’ looking preferences were not driven by interest in higher or faster jumps, and did not result merely from learning the relation between the height of the barrier and the height of the jump during habituation.
