# Supplementary material for: Moderated Online Data-Collection for Developmental Research: Methods and Replications
Source: Front Psychol. 2021 Nov 3;12:734398. doi: 10.3389/fpsyg.2021.734398 (PMC8595939; doi:10.3389/fpsyg.2021.734398)
Supplement: Supplementary file 1 [file Data_Sheet_1.PDF]

Online Methods Data Analysis

Aaron Chuey & Mike Frank

4/13/2021

Load the necessary libraries, try to remove the ones that aren't necessary because they cause intense conflicts. Curses on anyone who loads `plyr` unnecessarily.

```
library(tidyverse)
```

```
## -- Attaching packages --------------------------------------- tidyverse 1.3.0 --
```

```
## v ggplot2 3.3.3     v purrr   0.3.4
## v tibble  3.1.2     v dplyr   1.0.6
## v tidyr   1.0.0     v stringr 1.4.0
## v readr   1.3.1     v forcats 0.4.0
```

```
## Warning: package 'ggplot2' was built under R version 3.6.2
```

```
## Warning: package 'tibble' was built under R version 3.6.2
```

```
## Warning: package 'purrr' was built under R version 3.6.2
```

```
## Warning: package 'dplyr' was built under R version 3.6.2
```

```
## -- Conflicts ------------------------------------------ tidyverse_conflicts() --
## x dplyr::filter() masks stats::filter()
## x dplyr::lag()    masks stats::lag()
```

```
library(effsize)
```

```
## Warning: package 'effsize' was built under R version 3.6.2
```

```
library(effectsize)
```

```
## Warning: package 'effectsize' was built under R version 3.6.2
```

```
library(epitools)
```

```
##
## Attaching package: 'epitools'
```

```
## The following objects are masked from 'package:effectsize':
##
##     oddsratio, riskratio
```

```r
library(boot)
library(lme4)
```

```
## Loading required package: Matrix

##
## Attaching package: 'Matrix'

## The following objects are masked from 'package:tidyr':
##
##     expand, pack, unpack
```

```r
library(ggpubr)
```

```
## Warning: package 'ggpubr' was built under R version 3.6.2

## Registered S3 methods overwritten by 'car':
##   method                          from
##   influence.merMod                lme4
##   cooks.distance.influence.merMod lme4
##   dfbeta.influence.merMod         lme4
##   dfbetas.influence.merMod        lme4
```

```r
library(gridExtra)
```

```
##
## Attaching package: 'gridExtra'

## The following object is masked from 'package:dplyr':
##
##     combine
```

```r
library(plotrix)
library(EMAtools) #for deriving effect sizes
require(reshape2) # for woo/spelke
```

```
## Loading required package: reshape2

##
## Attaching package: 'reshape2'

## The following object is masked from 'package:tidyr':
##
##     smiths
```

```r
require(cowplot) # for big plot
```

```
## Loading required package: cowplot
```

```
##
## ********************************************************

## Note: As of version 1.0.0, cowplot does not change the

##   default ggplot2 theme anymore. To recover the previous

##   behavior, execute:
##   theme_set(theme_cowplot())

## ********************************************************

##
## Attaching package: 'cowplot'

## The following object is masked from 'package:ggpubr':
##
##     get_legend
```

```
library(metafor) # for meta-analysis?
```

```
## Loading 'metafor' package (version 2.4-0). For an overview
## and introduction to the package please type: help(metafor).
```

Individual Data analysis

Liu Spelke Data Analysis

Data reading

```
combined.data.long <- read.csv("data/liu_spelke_data.csv") %>%
  filter(subj != "P13") %>% # no data for test trials
  mutate_if(is.character,as.factor) %>%
  mutate(sample = recode(sample, "lab" = "In-Person",
                         "online" = "Online")) %>%
  mutate(trialtype = recode(trialtype,
                            "exp" = "Expected",
                            "unexp" = "Unexpected"))

sample.info <- combined.data.long %>%
  distinct(subj, .keep_all = TRUE) %>%
  group_by(sample) %>%
  summarize(mean = mean(agem), min=range(agem)[1], max=range(agem)[2], n=length(unique(subj)), f=sum(se

# average looking times
final.repl.data.avg <- combined.data.long %>%
  filter(!is.na(trialtype))%>%
  group_by(sample, subj, trialtype) %>%
  summarise(avg_look = mean(look, na.rm=TRUE))
```

```
## `summarise()` has grouped output by 'sample', 'subj'. You can override using the `.groups` argument.
```

In-person Results

```
test.avg <- final.repl.data.avg %>%
  filter(trialtype != "famhab") %>%
  mutate(loglook = log(avg_look))
test.avg$trialtype <- relevel(test.avg$trialtype, ref = "Expected")

orig.avg <- test.avg %>% filter(sample=="In-Person")

orig.m1 <- lmer(loglook ~ trialtype + (1|subj),
                data = orig.avg)
orig.m1.std <- lmer(scale(loglook) ~ trialtype + (1|subj),
                data = orig.avg)
```

Online Results

```
online.avg <- test.avg %>% filter(sample=="Online")

online.m1 <- lmer(loglook ~ trialtype + (1|subj),
                data = online.avg)

online.m1.cooks <- lmer(loglook ~ trialtype + (1|subj),
                data = online.avg %>% filter(subj != "P7-"))


online.m1.std <- lmer(scale(loglook) ~ trialtype + (1|subj),
                data = online.avg %>% filter(subj != "P7-"))
```

Woo Spelke Data Analysis

Experiment 1: Evaluations based on preferential reaching

```
# data organization
choice <- matrix(c(17, 5, 5, 19), ncol = 2, byrow = T)
rownames(choice) <- c("True Belief", "False Belief")
colnames(choice) <- c("Positive-Outcome", "Unhelpful-Outcome")

# visualize data
## using ggplot2
choice.d <- as.data.frame(choice)
choice.d$Condition <- c("True Belief", "False Belief")
choice.dm <- reshape2::melt(choice.d, id.vars = 'Condition')
choice.dm$Condition <- as.factor(choice.dm$Condition)

choice.dm$Condition <- relevel(choice.dm$Condition, "True Belief")
levels(choice.dm$variable)
```

```
## [1] "Positive-Outcome"  "Unhelpful-Outcome"
```

```r
choice.dm$variable <- relevel(choice.dm$variable, "Unhelpful-Outcome")
levels(choice.dm$variable) <- c("Unhelpful-Outcome", "Positive-Outcome")

choice.dm$variable.n <- choice.dm$variable
levels(choice.dm$variable.n) <- c("Unhelpful-\nOutcome", "Helpful-\nOutcome")

for (i in 1:nrow(choice.dm)) {
  if (choice.dm$Condition[i] == "True Belief") {
    if (choice.dm$variable[i] == "Positive-Outcome") {
      choice.dm$intention[i] <- "Helpful\nIntention"
    }
    else if (choice.dm$variable[i] == "Unhelpful-Outcome") {
      choice.dm$intention[i] <- "Irrelevant\nIntention"
    }
  }
  else if (choice.dm$Condition[i] == "False Belief") {
    if (choice.dm$variable[i] == "Positive-Outcome") {
      choice.dm$intention[i] <- "Irrelevant\nIntention"
    }
    else if (choice.dm$variable[i] == "Unhelpful-Outcome") {
      choice.dm$intention[i] <- "Helpful\nIntention"
    }
  }
}

for (i in 1:nrow(choice.dm)) {
  if (choice.dm$variable[i] == "Positive-Outcome") {
    choice.dm$outcome[i] <- "Positive\nOutcome"
  }
  else if (choice.dm$variable[i] == "Unhelpful-Outcome") {
    choice.dm$outcome[i] <- "Unhelpful\nOutcome"
  }
}

choice.dm$intention <- as.factor(choice.dm$intention)
choice.dm$intention <- relevel(choice.dm$intention, "Irrelevant\nIntention")
choice.dm$outcome <- as.factor(choice.dm$outcome)
choice.dm$outcome <- relevel(choice.dm$outcome, "Unhelpful\nOutcome")

ggplot(choice.dm, aes(intention, value)) + # grouped
  geom_bar(aes(fill = outcome), position = "dodge", stat="identity", color = "grey25") +
  facet_wrap(~Condition,  scales = "free_x") +
  scale_fill_manual(values = c("grey55", "grey100")) +
  labs(x = "Intention of Actor", fill = "Outcome", y = "Number of Infants Reaching") +
  theme(legend.position = "bottom") +
  ylim(c(0,25)) # Figure 2A; code for paper; new labels (intention and outcome both present)
```

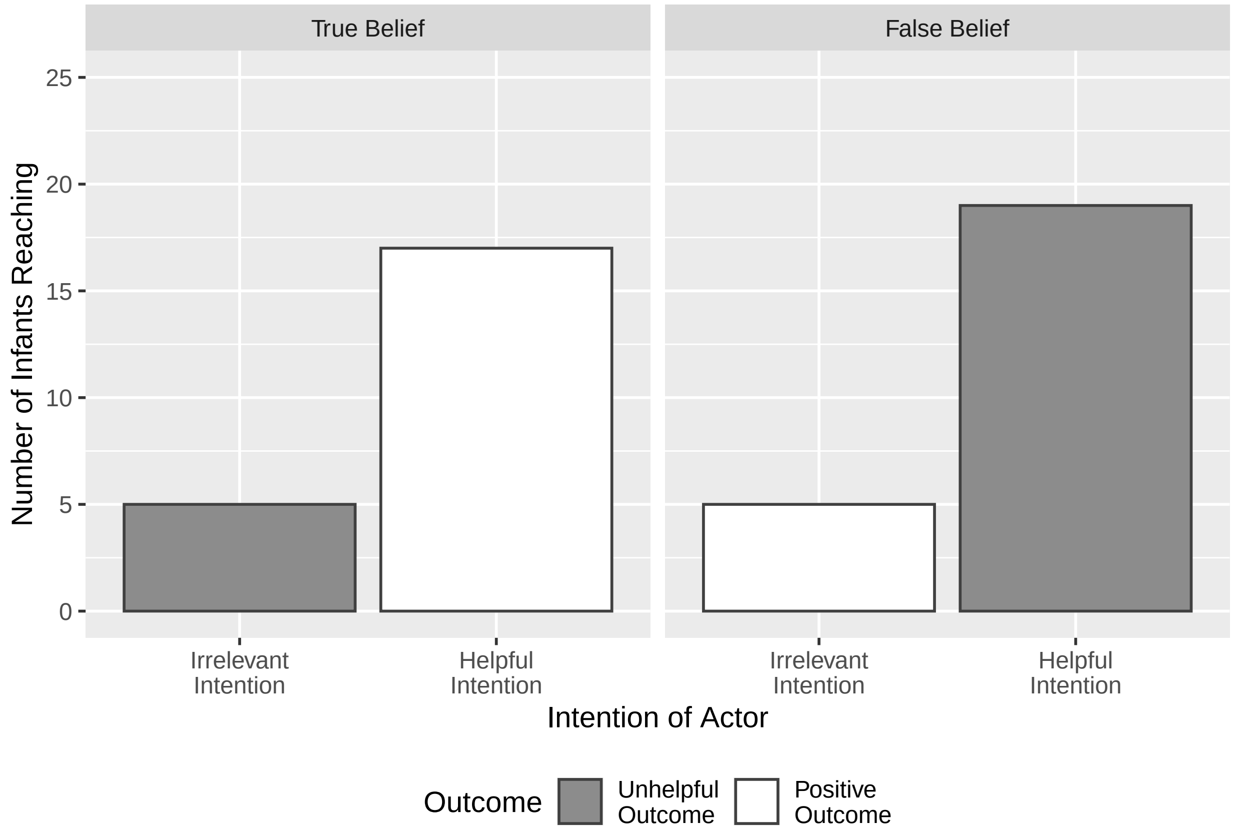


```
# binomial tests
## pref for Positive-Intention vs. Irrelevant-Intention (main text)
binom.test(17, n = 22, p = .5, alternative = "two.sided") # True Belief
```

```
##
##  Exact binomial test
##
## data:  17 and 22
## number of successes = 17, number of trials = 22, p-value = 0.0169
## alternative hypothesis: true probability of success is not equal to 0.5
## 95 percent confidence interval:
##  0.5462964 0.9217937
## sample estimates:
## probability of success
##              0.7727273
```

```
RR.TB <- 17/22/0.5 # RR = relative risk

binom.test(19, n = 24, p = .5, alternative = "two.sided") # False Belief
```

```
##
##  Exact binomial test
##
## data:  19 and 24
## number of successes = 19, number of trials = 24, p-value = 0.006611
## alternative hypothesis: true probability of success is not equal to 0.5
```

```
## 95 percent confidence interval:
##  0.5784872 0.9286814
## sample estimates:
## probability of success
##              0.7916667
```

```
RR.FB <- 19/24/0.5

# chi-square test: Difference in choice based on outcome, by condition (main text)
chisq.test(matrix(c(19,5,5,17), ncol = 2, byrow = T))
```

```
##
##  Pearson's Chi-squared test with Yates' continuity correction
##
## data:  matrix(c(19, 5, 5, 17), ncol = 2, byrow = T)
## X-squared = 12.478, df = 1, p-value = 0.0004117
```

```
oddsratio.wald.outcome <- epitools::oddsratio.wald(matrix(c(19,5,5,17), ncol = 2, byrow = T))$measure[2
# ES.h(17/22, 5/24) # Cohen's h
```

Experiment 2: Evaluations based on preferential looking

```
bh2zoom <- read.csv("data/woo_spelke_data.csv")
dim(bh2zoom)
```

```
## [1] 48 15
```

```
# participant details (main text)
summary(bh2zoom$Age.Decimal)
```

```
##    Min. 1st Qu.  Median    Mean 3rd Qu.    Max.
##   14.33   14.63   14.90   14.91   15.11   15.67
```

```
bh2zoom$Condition <- as.factor(bh2zoom$Condition)
levels(bh2zoom$Condition) <- c("False Belief", "True Belief")
by(bh2zoom$Age.Decimal, bh2zoom$Condition, summary)
```

```
## bh2zoom$Condition: False Belief
##    Min. 1st Qu.  Median    Mean 3rd Qu.    Max.
##   14.37   14.66   14.82   14.88   15.08   15.67
## ------------------------------------------------------------
## bh2zoom$Condition: True Belief
##    Min. 1st Qu.  Median    Mean 3rd Qu.    Max.
##   14.33   14.60   14.92   14.93   15.15   15.67
```

```
table(bh2zoom$Sex)
```

```
##
##  F  M
## 26 22
```

```r
by(bh2zoom$Sex, bh2zoom$Condition, table)
```

```
## bh2zoom$Condition: False Belief
##
##  F  M
## 11 13
## ------------------------------------------------------------
## bh2zoom$Condition: True Belief
##
##  F  M
## 15  9
```

```r
# data organization
names(bh2zoom)
```

```
##  [1] "Relabeled.Script"          "Script"
##  [3] "Subject.ID"                "Sex"
##  [5] "Age...Month"               "Age...Day"
##  [7] "Age.Decimal"               "Condition"
##  [9] "Fam.Box.Color"             "Pink.Actor.Show.Side"
## [11] "Pink.Actor.Order"          "Test.Actor.Old.Toy.Color"
## [13] "Pink.Actor.Choice.Side"    "ChoiceLeft"
## [15] "ChoiceRight"
```

```r
for (i in 1:nrow(bh2zoom)) {
  if (bh2zoom$Test.Actor.Old.Toy.Color[i] == "Pink") {
    if (bh2zoom$Pink.Actor.Choice.Side[i] == "Left") {
      bh2zoom$ChoicePositive0[i] <- bh2zoom$ChoiceLeft[i]
      bh2zoom$ChoiceUnhelpful0[i] <- bh2zoom$ChoiceRight[i]
    }
    else if (bh2zoom$Pink.Actor.Choice.Side[i] == "Right") {
      bh2zoom$ChoicePositive0[i] <- bh2zoom$ChoiceRight[i]
      bh2zoom$ChoiceUnhelpful0[i] <- bh2zoom$ChoiceLeft[i]
    }
  }
  else if (bh2zoom$Test.Actor.Old.Toy.Color[i] == "Yellow") {
    if (bh2zoom$Pink.Actor.Choice.Side[i] == "Right") {
      bh2zoom$ChoicePositive0[i] <- bh2zoom$ChoiceLeft[i]
      bh2zoom$ChoiceUnhelpful0[i] <- bh2zoom$ChoiceRight[i]
    }
    else if (bh2zoom$Pink.Actor.Choice.Side[i] == "Left") {
      bh2zoom$ChoicePositive0[i] <- bh2zoom$ChoiceRight[i]
      bh2zoom$ChoiceUnhelpful0[i] <- bh2zoom$ChoiceLeft[i]
    }
  }
}
by(bh2zoom$ChoicePositive0, bh2zoom$Condition, summary)
```

```
## bh2zoom$Condition: False Belief
##    Min. 1st Qu.  Median    Mean 3rd Qu.    Max.
##   2.650   7.808   9.725  10.415  11.723  23.680
## ------------------------------------------------------------
```

```
## bh2zoom$Condition: True Belief
##    Min. 1st Qu.  Median    Mean 3rd Qu.    Max.
##   4.070   9.793  12.500  13.257  16.617  22.500
```

```r
by(bh2zoom$ChoicePositiveO, bh2zoom$Condition, sd)
```

```
## bh2zoom$Condition: False Belief
## [1] 4.493414
## ------------------------------------------------------------
## bh2zoom$Condition: True Belief
## [1] 4.82981
```

```r
by(bh2zoom$ChoiceUnhelpfulO, bh2zoom$Condition, summary)
```

```
## bh2zoom$Condition: False Belief
##    Min. 1st Qu.  Median    Mean 3rd Qu.    Max.
##    5.44   11.75   14.47   13.55   16.28   19.53
## ------------------------------------------------------------
## bh2zoom$Condition: True Belief
##    Min. 1st Qu.  Median    Mean 3rd Qu.    Max.
##   3.300   6.505   8.905   9.378  11.607  18.240
```

```r
by(bh2zoom$ChoiceUnhelpfulO, bh2zoom$Condition, sd)
```

```
## bh2zoom$Condition: False Belief
## [1] 3.941788
## ------------------------------------------------------------
## bh2zoom$Condition: True Belief
## [1] 4.073916
```

```r
# convert to percentages
for (i in 1:nrow(bh2zoom)) {
  bh2zoom$pPositiveO[i] <- bh2zoom$ChoicePositiveO[i]/(bh2zoom$ChoiceUnhelpfulO[i] + bh2zoom$ChoicePosi
  bh2zoom$pUnhelpfulO[i] <- bh2zoom$ChoiceUnhelpfulO[i]/(bh2zoom$ChoiceUnhelpfulO[i] + bh2zoom$ChoicePos
}

for (i in 1:nrow(bh2zoom)) {
  if (bh2zoom$Condition[i] == "True Belief") {
    bh2zoom$pHelpfulI[i] <- bh2zoom$pPositiveO[i]
    bh2zoom$pIrrelevantI[i] <- bh2zoom$pUnhelpfulO[i]
  }
  else if (bh2zoom$Condition[i] == "False Belief") {
    bh2zoom$pIrrelevantI[i] <- bh2zoom$pPositiveO[i]
    bh2zoom$pHelpfulI[i] <- bh2zoom$pUnhelpfulO[i]
  }
}

by(bh2zoom$pPositiveO, bh2zoom$Condition, summary)
```

```
## bh2zoom$Condition: False Belief
##    Min. 1st Qu.  Median    Mean 3rd Qu.    Max.
```

```
##  0.1763  0.3371  0.4116  0.4293  0.4913  0.7949
## ------------------------------------------------------------
## bh2zoom$Condition: True Belief
##    Min. 1st Qu.  Median    Mean 3rd Qu.    Max.
##  0.3102  0.4985  0.6098  0.5829  0.6743  0.8721
```

```
by(bh2zoom$pPositiveO, bh2zoom$Condition, sd)
```

```
## bh2zoom$Condition: False Belief
## [1] 0.1465029
## ------------------------------------------------------------
## bh2zoom$Condition: True Belief
## [1] 0.1492586
```

```
by(bh2zoom$pUnhelpfulO, bh2zoom$Condition, summary)
```

```
## bh2zoom$Condition: False Belief
##    Min. 1st Qu.  Median    Mean 3rd Qu.    Max.
##  0.2051  0.5087  0.5884  0.5707  0.6629  0.8237
## ------------------------------------------------------------
## bh2zoom$Condition: True Belief
##    Min. 1st Qu.  Median    Mean 3rd Qu.    Max.
##  0.1279  0.3257  0.3902  0.4171  0.5015  0.6898
```

```
by(bh2zoom$pUnhelpfulO, bh2zoom$Condition, sd)
```

```
## bh2zoom$Condition: False Belief
## [1] 0.1465029
## ------------------------------------------------------------
## bh2zoom$Condition: True Belief
## [1] 0.1492586
```

```
by(bh2zoom$pHelpfulI, bh2zoom$Condition, summary)
```

```
## bh2zoom$Condition: False Belief
##    Min. 1st Qu.  Median    Mean 3rd Qu.    Max.
##  0.2051  0.5087  0.5884  0.5707  0.6629  0.8237
## ------------------------------------------------------------
## bh2zoom$Condition: True Belief
##    Min. 1st Qu.  Median    Mean 3rd Qu.    Max.
##  0.3102  0.4985  0.6098  0.5829  0.6743  0.8721
```

```
by(bh2zoom$pHelpfulI, bh2zoom$Condition, sd)
```

```
## bh2zoom$Condition: False Belief
## [1] 0.1465029
## ------------------------------------------------------------
## bh2zoom$Condition: True Belief
## [1] 0.1492586
```

```r
by(bh2zoom$pIrrelevantI, bh2zoom$Condition, summary)
```

```
## bh2zoom$Condition: False Belief
##    Min. 1st Qu.  Median    Mean 3rd Qu.    Max.
##  0.1763  0.3371  0.4116  0.4293  0.4913  0.7949
## ------------------------------------------------------------
## bh2zoom$Condition: True Belief
##    Min. 1st Qu.  Median    Mean 3rd Qu.    Max.
##  0.1279  0.3257  0.3902  0.4171  0.5015  0.6898
```

```r
by(bh2zoom$pIrrelevantI, bh2zoom$Condition, sd)
```

```
## bh2zoom$Condition: False Belief
## [1] 0.1465029
## ------------------------------------------------------------
## bh2zoom$Condition: True Belief
## [1] 0.1492586
```

```r
# visualize raw looking times (Figure 2B)
bh2zoomL <- gather(bh2zoom, ActorType, LT,
                  ChoicePositiveO, ChoiceUnhelpfulO)
head(bh2zoomL)
```

```
##   Relabeled.Script Script Subject.ID Sex Age...Month Age...Day Age.Decimal
## 1               1b      9        p25   F          15         9    15.30000
## 2               2b     10        p17   F          15         0    15.00000
## 3               3b      5        p26   F          14        10    14.33333
## 4               4b     14        p13   F          15        11    15.36667
## 5               5b      3        p22   M          14        18    14.60000
## 6               6b     12        p15   M          14        25    14.83333
##      Condition Fam.Box.Color Pink.Actor.Show.Side Pink.Actor.Order
## 1 False Belief          Blue                 Left                1
## 2 False Belief          Blue                 Left                1
## 3  True Belief         Green                 Left                2
## 4 False Belief         Green                 Left                2
## 5  True Belief          Blue                Right                2
## 6 False Belief          Blue                Right                2
##   Test.Actor.Old.Toy.Color Pink.Actor.Choice.Side ChoiceLeft ChoiceRight
## 1                     Pink                   Left      15.29       12.49
## 2                   Yellow                   Left       7.60       18.51
## 3                   Yellow                   Left       9.87       17.94
## 4                     Pink                   Left      10.61       11.84
## 5                     Pink                  Right      11.66       12.55
## 6                   Yellow                  Right      10.03       16.96
##   pPositiveO pUnhelpfulO pIrrelevantI pHelpfulI       ActorType    LT
## 1  0.5503960   0.4496040    0.5503960 0.4496040 ChoicePositiveO 15.29
## 2  0.7089238   0.2910762    0.7089238 0.2910762 ChoicePositiveO 18.51
## 3  0.6450917   0.3549083    0.3549083 0.6450917 ChoicePositiveO 17.94
## 4  0.4726058   0.5273942    0.4726058 0.5273942 ChoicePositiveO 10.61
## 5  0.5183808   0.4816192    0.4816192 0.5183808 ChoicePositiveO 12.55
## 6  0.3716191   0.6283809    0.3716191 0.6283809 ChoicePositiveO 10.03
```

```r
hist(bh2zoomL$LT)
```

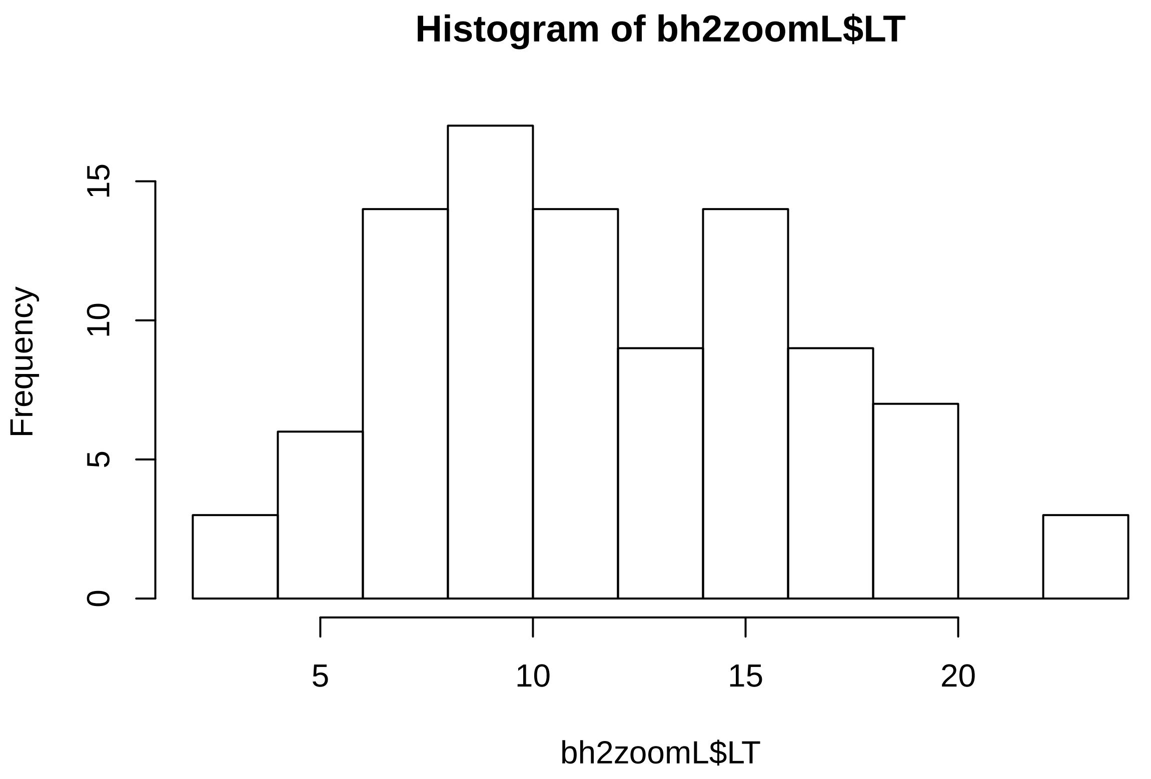


```r
class(bh2zoomL$ActorType)
```

```
## [1] "character"
```

```r
bh2zoomL$ActorType <- as.factor(bh2zoomL$ActorType)
bh2zoomL$Condition <- as.factor(bh2zoomL$Condition)
levels(bh2zoomL$ActorType) <- c("Positive-Outcome", "Unhelpful-Outcome")
bh2zoomL$ActorType <- relevel(bh2zoomL$ActorType, "Unhelpful-Outcome")
bh2zoomL$Outcome <- bh2zoomL$ActorType
levels(bh2zoomL$Outcome) <- c("Unhelpful\nOutcome", "Positive\nOutcome") # note: this is reversed from
bh2zoomL$Condition <- relevel(bh2zoomL$Condition, "True Belief")

table(bh2zoomL$ActorType)
```

```
##
## Unhelpful-Outcome  Positive-Outcome
##                48                48
```

```r
for (i in 1:nrow(bh2zoomL)) {
  if (bh2zoomL$Condition[i] == "True Belief") {
    if (bh2zoomL$Outcome[i] == "Unhelpful\nOutcome") {
      bh2zoomL$Intention[i] <- "Irrelevant\nIntention"
    }
    else if (bh2zoomL$Outcome[i] == "Positive\nOutcome") {
      bh2zoomL$Intention[i] <- "Helpful\nIntention"
```

```
    }
  }
  else if (bh2zoomL$Condition[i] == "False Belief") {
      if (bh2zoomL$Outcome[i] == "Positive\nOutcome") {
        bh2zoomL$Intention[i] <- "Irrelevant\nIntention"
      }
      else if (bh2zoomL$Outcome[i] == "Unhelpful\nOutcome") {
        bh2zoomL$Intention[i] <- "Helpful\nIntention"
      }
  }
}
bh2zoomL$Intention <- as.factor(bh2zoomL$Intention)
bh2zoomL$Intention <- relevel(bh2zoomL$Intention, "Irrelevant\nIntention")


ggplot(data = dplyr::filter(bh2zoomL), aes(x = Intention, y = LT, fill = Outcome)) +
  geom_boxplot(outlier.shape = NA) +
  facet_grid(~Condition) +
  geom_point(alpha = 0.25, show.legend = F) +
  geom_line(aes(group = Subject.ID), alpha = .15, linetype = 1) +
  stat_summary(fun = mean, geom = "point", size = 3, color = "red", shape = 18, show.legend = F) +
  theme(legend.position = "bottom") +
  scale_fill_manual(values = c("grey55", "grey100")) +
  ylab("Time (s) Looking") + xlab ("Intention of Actor") + ylim(0, 30) # Figure 2B
```

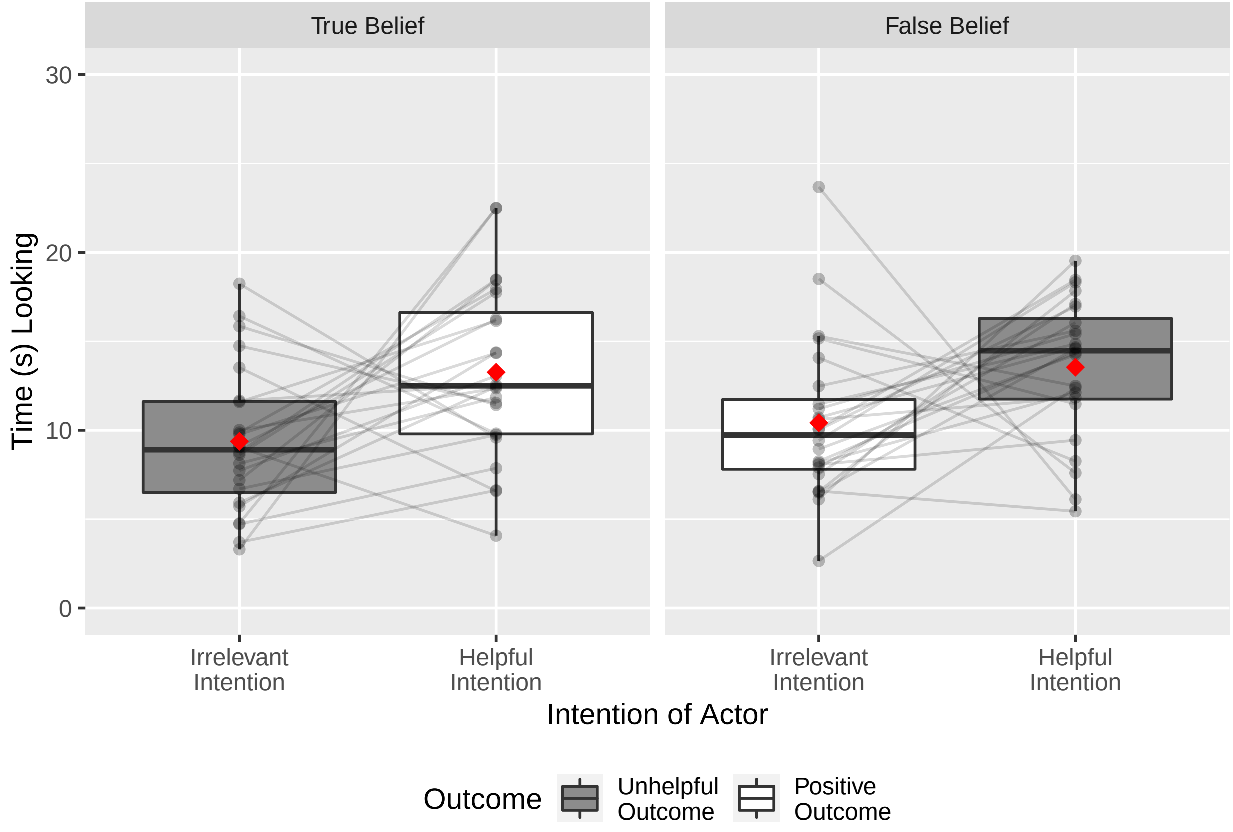

```r
# one sample t-test (proportionate looking)
## pref for Irrelevant-Intention vs. Positive-Intention (main text)
by(bh2zoom$pHelpfulI, bh2zoom$Condition, t.test, mu = 0.5, alternative = "two.sided")
```

```
## bh2zoom$Condition: False Belief
##
##  One Sample t-test
##
## data:  dd[x, ]
## t = 2.3658, df = 23, p-value = 0.0268
## alternative hypothesis: true mean is not equal to 0.5
## 95 percent confidence interval:
##  0.5088866 0.6326121
## sample estimates:
## mean of x
## 0.5707493
##
## ------------------------------------------------------------
## bh2zoom$Condition: True Belief
##
##  One Sample t-test
##
## data:  dd[x, ]
## t = 2.7197, df = 23, p-value = 0.01222
## alternative hypothesis: true mean is not equal to 0.5
## 95 percent confidence interval:
##  0.5198361 0.6458888
## sample estimates:
## mean of x
## 0.5828624
```

```r
by(bh2zoom$pHelpfulI, bh2zoom$Condition, lsr::cohensD, mu = 0.5)
```

```
## bh2zoom$Condition: False Belief
## [1] 0.482921
## ------------------------------------------------------------
## bh2zoom$Condition: True Belief
## [1] 0.5551601
```

```r
# two sample t-test: Difference in proportionate looking based on outcome, by condition (main text)
t.test(pPositiveO ~ Condition, data = bh2zoom, alternative = "two.sided")
```

```
##
##  Welch Two Sample t-test
##
## data:  pPositiveO by Condition
## t = -3.5982, df = 45.984, p-value = 0.0007807
## alternative hypothesis: true difference in means is not equal to 0
## 95 percent confidence interval:
##  -0.23954580 -0.06767772
## sample estimates:
## mean in group False Belief  mean in group True Belief
##                  0.4292507                  0.5828624
```

```
twosamplet.outcome <- t.test(pPositive0 ~ Condition, data = bh2zoom, alternative = "two.sided")
twosampled.outcome <- effsize::cohen.d(pPositive0 ~ Condition, data = bh2zoom)$estimate
```

Leonard Data Analysis

Data reading.

```
leonard_data <- read_csv("data/leonard_data.csv") %>%
  filter(include == 1) %>%
  mutate(version = recode(version, "in_person" = "In-Person", "online" = "Online"))
```

```
## Parsed with column specification:
## cols(
##   subject = col_character(),
##   date = col_character(),
##   gender = col_character(),
##   age_months = col_double(),
##   age = col_double(),
##   version = col_character(),
##   practice = col_double(),
##   test_question = col_double(),
##   inclusion_q = col_character(),
##   include = col_double(),
##   exclude_reason = col_character()
## )
```

```
#split data into in person and online versions
leonard_inperson <- filter(leonard_data, version == "In-Person")
leonard_online <- filter(leonard_data, version == "Online")
```

In person results

```
#are children above chance at critical question?
model<-binom.test(sum(leonard_inperson$test_question),20,p=.5)
model #significant
```

```
##
##  Exact binomial test
##
## data:  sum(leonard_inperson$test_question) and 20
## number of successes = 18, number of trials = 20, p-value = 0.0004025
## alternative hypothesis: true probability of success is not equal to 0.5
## 95 percent confidence interval:
##  0.6830173 0.9876515
## sample estimates:
## probability of success
##                    0.9
```

```
#get bootstrapped CI
ProportionFunction<-function(data,indices){return(sum(data[indices])/length(data[indices]))}
table(leonard_inperson$test_question)
```

```
##
##  0  1
##  2 18
```

```
##95% ci
exp1<-c(rep(1,18),rep(0,2))
resultsexp1 <- boot::boot(data=exp1, statistic=ProportionFunction, R=500000)
boot::boot.ci(resultsexp1,type="basic")
```

```
## BOOTSTRAP CONFIDENCE INTERVAL CALCULATIONS
## Based on 500000 bootstrap replicates
##
## CALL :
## boot::boot.ci(boot.out = resultsexp1, type = "basic")
##
## Intervals :
## Level      Basic
## 95%   ( 0.80,  1.05 )
## Calculations and Intervals on Original Scale
```

```
##included children who got "which tower was better" question wrong
d <- filter(leonard_data, version == "In-Person" & exclude_reason != "practice")

#are children above chance at critical question?
model <- binom.test(sum(d$test_question),27,p=.5)
model
```

```
##
##  Exact binomial test
##
## data:  sum(d$test_question) and 27
## number of successes = 0, number of trials = 27, p-value = 1.49e-08
## alternative hypothesis: true probability of success is not equal to 0.5
## 95 percent confidence interval:
##  0.0000000 0.1277029
## sample estimates:
## probability of success
##                      0
```

```
##95% ci
table(d$test_question)
```

```
## < table of extent 0 >
```

```
exp1<-c(rep(1,20),rep(0,7))
resultsexp1 <- boot(data=exp1, statistic=ProportionFunction, R=50000)
boot.ci(resultsexp1,type="basic")
```

```
## BOOTSTRAP CONFIDENCE INTERVAL CALCULATIONS
## Based on 50000 bootstrap replicates
##
## CALL :
## boot.ci(boot.out = resultsexp1, type = "basic")
##
## Intervals :
## Level      Basic
## 95%   ( 0.5926,  0.9259 )
## Calculations and Intervals on Original Scale
```

Online results

```
#are children above chance at critical question?
model<-binom.test(sum(leonard_online$test_question),20,p=.5)
model #significant
```

```
##
##  Exact binomial test
##
## data:  sum(leonard_online$test_question) and 20
## number of successes = 17, number of trials = 20, p-value = 0.002577
## alternative hypothesis: true probability of success is not equal to 0.5
## 95 percent confidence interval:
##  0.6210732 0.9679291
## sample estimates:
## probability of success
##                  0.85
```

```
#get bootstrapped CI
ProportionFunction<-function(data,indices){return(sum(data[indices])/length(data[indices]))}
table(leonard_online$test_question)
```

```
##
##  0  1
##  3 17
```

```
##95% ci
exp1<-c(rep(1,17),rep(0,3))
resultsexp1 <- boot::boot(data=exp1, statistic=ProportionFunction, R=50000)
boot::boot.ci(resultsexp1,type="basic")
```

```
## BOOTSTRAP CONFIDENCE INTERVAL CALCULATIONS
## Based on 50000 bootstrap replicates
##
## CALL :
## boot::boot.ci(boot.out = resultsexp1, type = "basic")
##
## Intervals :
## Level      Basic
## 95%   ( 0.7,  1.0 )
## Calculations and Intervals on Original Scale
```

Asaba Wu Data Analysis

Data reading.

```
asaba_data = read_csv("data/asaba_wu_data.csv")
```

```
## Warning: Missing column names filled in: 'X1' [1]
```

```
## Parsed with column specification:
## cols(
##   X1 = col_double(),
##   id = col_character(),
##   location = col_character(),
##   age = col_double(),
##   activity = col_character(),
##   outcome = col_character(),
##   question = col_character(),
##   chose_surprise = col_double(),
##   correct = col_double()
## )
```

```
asaba_data = asaba_data %>%
  mutate(location = recode(location, "in-person" = "In-Person",
                          "online" = "Online")) %>%
  mutate(outcome = recode(outcome, "fail" = "Fail",
                          "success" = "Success"))
emo_inperson = asaba_data %>%
  filter(location == "In-Person") %>%
  filter(age > 5.99)
emo_online = filter(asaba_data, location == "Online")
```

In person

```
inperson_glm = glmer(chose_surprise ~ outcome * age + activity + (1|id),
                    data = emo_inperson, family = "binomial")
```

```
## Warning in checkConv(attr(opt, "derivs"), opt$par, ctrl = control$checkConv, :
## Model failed to converge with max|grad| = 0.0420008 (tol = 0.001, component 1)
```

```
summary(inperson_glm)
```

```
## Generalized linear mixed model fit by maximum likelihood (Laplace
##   Approximation) [glmerMod]
##  Family: binomial  ( logit )
## Formula: chose_surprise ~ outcome * age + activity + (1 | id)
##    Data: emo_inperson
##
##      AIC      BIC   logLik deviance df.resid
##     64.5     86.3    -24.3     48.5      104
```

```
##
## Scaled residuals:
##     Min      1Q  Median      3Q     Max
## -1.6451 -0.0937 -0.0372  0.1153 15.2453
##
## Random effects:
##  Groups Name        Variance Std.Dev.
##  id     (Intercept) 3.588    1.894
## Number of obs: 112, groups:  id, 14
##
## Fixed effects:
##                    Estimate Std. Error z value Pr(>|z|)
## (Intercept)        -14.9529     7.7463  -1.930   0.0536 .
## outcomeSuccess      13.1479     9.4011   1.399   0.1619
## age                  2.2839     1.0471   2.181   0.0292 *
## activitymath        -0.7210     1.2175  -0.592   0.5537
## activityspelling    -1.4221     1.2541  -1.134   0.2568
## activitythrow       -0.7181     1.2174  -0.590   0.5553
## outcomeSuccess:age  -2.6440     1.2978  -2.037   0.0416 *
## ---
## Signif. codes:  0 '***' 0.001 '**' 0.01 '*' 0.05 '.' 0.1 ' ' 1
##
## Correlation of Fixed Effects:
##             (Intr) otcmSc age    actvtym actvtys actvtyt
## outcomSccss -0.501
## age         -0.989  0.514
## activitymth -0.013 -0.027 -0.074
## actvtysplln  0.071 -0.067 -0.164  0.528
## activtythrw -0.014 -0.027 -0.073  0.517   0.528
## otcmSccss:g  0.539 -0.987 -0.562  0.054   0.114   0.054
## convergence code: 0
## Model failed to converge with max|grad| = 0.0420008 (tol = 0.001, component 1)
```

Online

```r
online_glm = glmer(chose_surprise ~ outcome * age + activity + (1|id),
                   data = emo_online, family = "binomial")
summary(online_glm)
```

```
## Generalized linear mixed model fit by maximum likelihood (Laplace
##   Approximation) [glmerMod]
##  Family: binomial  ( logit )
## Formula: chose_surprise ~ outcome * age + activity + (1 | id)
##    Data: emo_online
##
##      AIC      BIC   logLik deviance df.resid
##    351.0    382.1   -167.5    335.0      352
##
## Scaled residuals:
##      Min       1Q   Median       3Q      Max
## -12.6094  -0.3598   0.0973   0.3308   3.0009
##
```

```
## Random effects:
##  Groups Name        Variance Std.Dev.
##  id     (Intercept) 4.578    2.14
## Number of obs: 360, groups:  id, 90
##
## Fixed effects:
##                       Estimate Std. Error z value Pr(>|z|)
## (Intercept)         -2.147e+00  3.092e+00  -0.694  0.48747
## outcomeSuccess       4.454e+00  3.210e+00   1.387  0.16533
## age                  7.051e-01  4.122e-01   1.711  0.08716 .
## activitymath        -2.312e-05  4.430e-01   0.000  0.99996
## activityspelling    -7.186e-01  5.045e-01  -1.424  0.15431
## activitythrow       -3.905e-01  5.014e-01  -0.779  0.43606
## outcomeSuccess:age -1.157e+00  4.386e-01  -2.638  0.00834 **
## ---
## Signif. codes:  0 '***' 0.001 '**' 0.01 '*' 0.05 '.' 0.1 ' ' 1
##
## Correlation of Fixed Effects:
##             (Intr) otcmSc age    actvtym actvtys actvtyt
## outcomSccss -0.616
## age         -0.983  0.628
## activitymth -0.072  0.000  0.000
## actvtysplln -0.079 -0.029 -0.024  0.439
## activtythrw -0.083 -0.020 -0.011  0.442   0.564
## otcmSccss:g  0.601 -0.985 -0.635  0.000   0.067   0.047
```

Meta Analysis

Effect size computation

Leonard data: binomial test (choice) + 95% CI. Will run binomial tests on both online and in person experiments

```r
#unified model
leonard_mod <- glm(test_question ~ version,
                   data = leonard_data)

# convert odds ratio to d
leonard_diff_es <- logoddsratio_to_d(leonard_mod[1]$coefficients[2])

#in person model
leonard_test_inperson <- binom.test(sum(leonard_inperson$test_question),20,p=.5)

leonard_es_inperson <- effectsize::oddsratio_to_d(mean(leonard_inperson$test_question)/(1-mean(leonard_i

leonard_es_online <- effectsize::oddsratio_to_d(mean(leonard_online$test_question)/(1-mean(leonard_onlin

leonard_ma <- tribble(~experiment, ~context, ~n, ~es, ~var,
                      "Study 1", "In-Person",
                      length(leonard_inperson$test_question), leonard_es_inperson,
                      compute.es::des(d=leonard_es_inperson, n.1 = 10, n.2 = 10)$var.d,
                      "Study 1", "Online",
```

```r
                      length(leonard_online$test_question), leonard_es_online,
                      compute.es::des(d=leonard_es_online, n.1 = 10, n.2 = 10)$var.d)
```

```
## Mean Differences ES:
##
##  d [ 95 %CI] = 1.21 [ 0.26 , 2.16 ]
##   var(d) = 0.24
##   p-value(d) = 0.02
##   U3(d) = 88.71 %
##   CLES(d) = 80.42 %
##   Cliff's Delta = 0.61
##
##  g [ 95 %CI] = 1.16 [ 0.25 , 2.07 ]
##   var(g) = 0.22
##   p-value(g) = 0.02
##   U3(g) = 87.7 %
##   CLES(g) = 79.4 %
##
##  Correlation ES:
##
##  r [ 95 %CI] = 0.52 [ 0.1 , 0.78 ]
##   var(r) = 0.02
##   p-value(r) = 0.03
##
##  z [ 95 %CI] = 0.57 [ 0.1 , 1.05 ]
##   var(z) = 0.06
##   p-value(z) = 0.03
##
##  Odds Ratio ES:
##
##  OR [ 95 %CI] = 9 [ 1.6 , 50.74 ]
##   p-value(OR) = 0.02
##
##  Log OR [ 95 %CI] = 2.2 [ 0.47 , 3.93 ]
##   var(lOR) = 0.78
##   p-value(Log OR) = 0.02
##
##  Other:
##
##  NNT = 2.25
##  Total N = 20Mean Differences ES:
##
##  d [ 95 %CI] = 0.96 [ 0.03 , 1.88 ]
##   var(d) = 0.22
##   p-value(d) = 0.06
##   U3(d) = 83.05 %
##   CLES(d) = 75.06 %
##   Cliff's Delta = 0.5
##
##  g [ 95 %CI] = 0.92 [ 0.03 , 1.8 ]
##   var(g) = 0.2
##   p-value(g) = 0.06
##   U3(g) = 82.01 %
```

```
##    CLES(g) = 74.14 %
##
##   Correlation ES:
##
##   r [ 95 %CI] = 0.43 [ -0.01 , 0.73 ]
##    var(r) = 0.03
##    p-value(r) = 0.07
##
##   z [ 95 %CI] = 0.46 [ -0.01 , 0.94 ]
##    var(z) = 0.06
##    p-value(z) = 0.07
##
##   Odds Ratio ES:
##
##   OR [ 95 %CI] = 5.67 [ 1.06 , 30.35 ]
##    p-value(OR) = 0.06
##
##   Log OR [ 95 %CI] = 1.73 [ 0.06 , 3.41 ]
##    var(lOR) = 0.73
##    p-value(Log OR) = 0.06
##
##   Other:
##
##   NNT = 2.89
##   Total N = 20
```

Asaba Wu data: mixed effects logistic regression (choice), outcome was main variable of interest + age (and interaction). Will keep this analysis for both studies. Effect size (d) for each fixed effect was derived via EMAtools package

```
asaba_data$age.c <- scale(asaba_data$age, center = TRUE, scale = FALSE)
#combined model
asaba_mod <- glmer(chose_surprise ~ outcome * age.c * location +
                     + (1|id),
                   family = "binomial", data = asaba_data)
```

```
## Warning in checkConv(attr(opt, "derivs"), opt$par, ctrl = control$checkConv, :
## Model failed to converge with max|grad| = 0.00193749 (tol = 0.001, component 1)
```

```
#in person model
asaba_test_inperson <- glmer(chose_surprise ~ outcome * age.c + (1|id),
                   family = "binomial", data = asaba_data %>% filter(location == "In-Person"))
#online model
asaba_test_online <- glmer(chose_surprise ~ outcome * age.c + (1|id),
                   family = "binomial", data = asaba_data %>% filter(location == "Online"))

#effect sizes of in person model
asaba_es_inperson = lme.dscore(asaba_test_inperson,
                               data=asaba_data %>% filter(location == "In-Person"),
                               type="lme4")
#effect sizes of online moel
asaba_es_online = lme.dscore(asaba_test_online,
                             data=asaba_data %>% filter(location == "Online"),
```

```
                              type="lme4")

n_inperson <- length(unique(filter(asaba_data, location == "In-Person")$id))
n_online <- length(unique(filter(asaba_data, location == "Online")$id))

# note:ES are negative, so we reverse them
aw_ma <- tribble(~experiment, ~context, ~n, ~es, ~var,
                 "Study 2", "In-Person",
                 n_inperson,
                 -asaba_es_inperson$d[1],
                 compute.es::des(d=-asaba_es_inperson$d[1],
                                 n.1 = n_inperson/2, n.2 = n_inperson/2)$var.d,
                 "Study 2", "Online",
                 n_online, -asaba_es_online$d[1],
                 compute.es::des(d=-asaba_es_online$d[1],
                                 n.1 = n_online/2, n.2 = n_online/2)$var.d)
```

```
## Mean Differences ES:
##
##  d [ 95 %CI] = 1.38 [ 0.56 , 2.21 ]
##   var(d) = 0.18
##   p-value(d) = 0
##   U3(d) = 91.68 %
##   CLES(d) = 83.61 %
##   Cliff's Delta = 0.67
##
##  g [ 95 %CI] = 1.34 [ 0.54 , 2.14 ]
##   var(g) = 0.17
##   p-value(g) = 0
##   U3(g) = 91.04 %
##   CLES(g) = 82.89 %
##
##  Correlation ES:
##
##  r [ 95 %CI] = 0.57 [ 0.25 , 0.78 ]
##   var(r) = 0.01
##   p-value(r) = 0
##
##  z [ 95 %CI] = 0.65 [ 0.25 , 1.04 ]
##   var(z) = 0.04
##   p-value(z) = 0
##
##  Odds Ratio ES:
##
##  OR [ 95 %CI] = 12.3 [ 2.76 , 54.91 ]
##   p-value(OR) = 0
##
##  Log OR [ 95 %CI] = 2.51 [ 1.01 , 4.01 ]
##   var(lOR) = 0.58
##   p-value(Log OR) = 0
##
##  Other:
##
```

```
##  NNT = 1.98
##  Total N = 28Mean Differences ES:
##
##  d [ 95 %CI] = 1.47 [ 1 , 1.93 ]
##   var(d) = 0.06
##   p-value(d) = 0
##   U3(d) = 92.88 %
##   CLES(d) = 85.02 %
##   Cliff's Delta = 0.7
##
##  g [ 95 %CI] = 1.45 [ 0.99 , 1.92 ]
##   var(g) = 0.06
##   p-value(g) = 0
##   U3(g) = 92.71 %
##   CLES(g) = 84.82 %
##
##  Correlation ES:
##
##  r [ 95 %CI] = 0.59 [ 0.44 , 0.71 ]
##   var(r) = 0
##   p-value(r) = 0
##
##  z [ 95 %CI] = 0.68 [ 0.47 , 0.89 ]
##   var(z) = 0.01
##   p-value(z) = 0
##
##  Odds Ratio ES:
##
##  OR [ 95 %CI] = 14.31 [ 6.15 , 33.3 ]
##   p-value(OR) = 0
##
##  Log OR [ 95 %CI] = 2.66 [ 1.82 , 3.51 ]
##   var(lOR) = 0.19
##   p-value(Log OR) = 0
##
##  Other:
##
##  NNT = 1.87
##  Total N = 90
```

Woo Spelke data: for exp 1, binomial test (choice) for each condition, chi square for comparison between conditions; for exp 2, one sample t's for each condition, two sample t's for comparison between conditions. Will run binomial tests + two sample t's on both online + in person experiments.

```
values <- choice.dm$value
chi2 <- chisq.test(matrix(values, nrow=2))

woospelke1_es <- esc::esc_chisq(chisq = chi2$statistic, totaln = sum(values),
                                es.type = "d")
```

For exp2

```r
bh2zoomL %>%
  group_by(Subject.ID, Condition) %>%
  summarise(LT_diff = LT[Outcome == "Positive\nOutcome"] - LT[Outcome == "Unhelpful\nOutcome"]) %>%
  group_by(Condition) %>%
  summarise(d = mean(LT_diff) / sd(LT_diff))
```

```
## `summarise()` has grouped output by 'Subject.ID'. You can override using the `.groups` argument.
```

```
## # A tibble: 2 x 2
##   Condition         d
##   <fct>         <dbl>
## 1 True Belief   0.537
## 2 False Belief -0.432
```

```r
woospelke2_mod <- lmer(LT ~ Condition * Outcome + (1 | Subject.ID),
     data = bh2zoomL)
```

```
## boundary (singular) fit: see ?isSingular
```

```r
woospelke2_es <- lme.dscore(woospelke2_mod, data = bh2zoomL, type = "lme4") %>%
  slice(3)
```

```
## boundary (singular) fit: see ?isSingular
```

```r
# what's the right ES for this interaction term... derive from an ANOVA?
```

```r
ws_ma <- tribble(~experiment, ~context, ~n, ~es, ~var,
                 "Study 3", "In-Person",
                 sum(choice.dm$value) , woospelke1_es$es, woospelke1_es$var,
                 "Study 3", "Online", length(unique(bh2zoomL$Subject.ID)),
                 -woospelke2_es$d[1], # have to flip sign on interaction
                 compute.es::des(d=-woospelke2_es$d[1], n.1 = 24, n.2 = 24)$var.d)
```

```
## Mean Differences ES:
##
##  d [ 95 %CI] = 0.82 [ 0.23 , 1.41 ]
##   var(d) = 0.09
##   p-value(d) = 0.01
##   U3(d) = 79.49 %
##   CLES(d) = 71.98 %
##   Cliff's Delta = 0.44
##
##  g [ 95 %CI] = 0.81 [ 0.23 , 1.39 ]
##   var(g) = 0.09
##   p-value(g) = 0.01
##   U3(g) = 79.1 %
##   CLES(g) = 71.66 %
##
##  Correlation ES:
##
```

```
##   r [ 95 %CI] = 0.38 [ 0.11 , 0.6 ]
##    var(r) = 0.01
##    p-value(r) = 0.01
##
##   z [ 95 %CI] = 0.4 [ 0.11 , 0.69 ]
##    var(z) = 0.02
##    p-value(z) = 0.01
##
##   Odds Ratio ES:
##
##   OR [ 95 %CI] = 4.45 [ 1.53 , 12.97 ]
##    p-value(OR) = 0.01
##
##   Log OR [ 95 %CI] = 1.49 [ 0.42 , 2.56 ]
##    var(lOR) = 0.3
##    p-value(Log OR) = 0.01
##
##   Other:
##
##   NNT = 3.42
##   Total N = 48
```

Liu Spelke data: looking time by trial type, mixed effects linear model; effect size (d) of trial type was derived via EMAtools package

```
liu_test_inperson <- lmer(scale(loglook) ~ trialtype + (1|subj),
                data = orig.avg)
liu_test_online <- lmer(scale(loglook) ~ trialtype + (1|subj),
                data = online.avg %>% filter(subj != "P7-"))
liu_es_inperson <- lme.dscore(liu_test_inperson,
                              data=orig.avg,
                              type="lme4")
liu_es_online <- lme.dscore(liu_test_inperson,
                            data=online.avg %>% filter(subj != "P7-"),
                            type="lme4")

ls_ma <- tribble(~experiment, ~context, ~n, ~es, ~var,
                 "Study 4", "In-Person",
                 length(unique(orig.avg$subj)), liu_es_inperson$d[1],
                 compute.es::des(d=liu_es_inperson$d[1], n.1 = length(unique(orig.avg$subj))/2,
                                 n.2 = length(unique(orig.avg$subj))/2)$var.d,
                 "Study 4", "Online",
                 length(unique(online.avg$subj)), liu_es_online$d[1],
                 compute.es::des(d=liu_es_online$d[1], n.1 = length(unique(online.avg$subj))/2,
                                 n.2 = length(unique(online.avg$subj))/2)$var.d)
```

```
## Mean Differences ES:
##
##   d [ 95 %CI] = 1.43 [ 0.45 , 2.41 ]
##    var(d) = 0.25
##    p-value(d) = 0.01
##    U3(d) = 92.32 %
##    CLES(d) = 84.35 %
```

```
##   Cliff's Delta = 0.69
##
##  g [ 95 %CI] = 1.37 [ 0.43 , 2.31 ]
##   var(g) = 0.23
##   p-value(g) = 0.01
##   U3(g) = 91.41 %
##   CLES(g) = 83.3 %
##
##  Correlation ES:
##
##  r [ 95 %CI] = 0.58 [ 0.19 , 0.81 ]
##   var(r) = 0.02
##   p-value(r) = 0.01
##
##  z [ 95 %CI] = 0.66 [ 0.19 , 1.14 ]
##   var(z) = 0.06
##   p-value(z) = 0.01
##
##  Odds Ratio ES:
##
##  OR [ 95 %CI] = 13.3 [ 2.24 , 78.93 ]
##   p-value(OR) = 0.01
##
##  Log OR [ 95 %CI] = 2.59 [ 0.81 , 4.37 ]
##   var(lOR) = 0.83
##   p-value(Log OR) = 0.01
##
##  Other:
##
##  NNT = 1.92
##  Total N = 20Mean Differences ES:
##
##  d [ 95 %CI] = 1.09 [ 0.28 , 1.9 ]
##   var(d) = 0.17
##   p-value(d) = 0.01
##   U3(d) = 86.17 %
##   CLES(d) = 77.91 %
##   Cliff's Delta = 0.56
##
##  g [ 95 %CI] = 1.05 [ 0.27 , 1.84 ]
##   var(g) = 0.16
##   p-value(g) = 0.01
##   U3(g) = 85.43 %
##   CLES(g) = 77.22 %
##
##  Correlation ES:
##
##  r [ 95 %CI] = 0.48 [ 0.12 , 0.73 ]
##   var(r) = 0.02
##   p-value(r) = 0.02
##
##  z [ 95 %CI] = 0.52 [ 0.12 , 0.92 ]
##   var(z) = 0.04
##   p-value(z) = 0.02
```

```
##
##  Odds Ratio ES:
##
##  OR [ 95 %CI] = 7.19 [ 1.66 , 31.17 ]
##   p-value(OR) = 0.01
##
##  Log OR [ 95 %CI] = 1.97 [ 0.51 , 3.44 ]
##   var(lOR) = 0.56
##   p-value(Log OR) = 0.01
##
##  Other:
##
##  NNT = 2.52
##  Total N = 27
```

Meta-analysis

Get everything into a tibble.

```
ma_data <- bind_rows(leonard_ma, aw_ma, ws_ma, ls_ma)
ma_data
```

```
## # A tibble: 8 x 5
##   experiment context       n    es   var
##   <chr>      <chr>     <dbl> <dbl> <dbl>
## 1 Study 1    In-Person    20 1.21  0.24
## 2 Study 1    Online       20 0.956 0.22
## 3 Study 2    In-Person    28 1.38  0.18
## 4 Study 2    Online       90 1.47  0.06
## 5 Study 3    In-Person    46 1.22  0.119
## 6 Study 3    Online       48 0.823 0.09
## 7 Study 4    In-Person    20 1.43  0.25
## 8 Study 4    Online       27 1.09  0.17
```

Our approach is random-effects meta-regression with study-wise grouping. We're going to weight by N instead of ES variance because of the differences in ES.

```
# escalc(yi = es, ni = n, data = ma_data)

ma_mod <- rma.mv(yi = es, V = var, random = ~ 1 |experiment,
                 mods = ~context, slab = experiment, data = ma_data)
summary(ma_mod)
```

```
##
## Multivariate Meta-Analysis Model (k = 8; method: REML)
##
##   logLik  Deviance       AIC       BIC      AICc
##  -1.5149    3.0298    9.0298    8.4051   21.0298
##
## Variance Components:
##
##              estim    sqrt  nlvls  fixed      factor
```

```
## sigma^2    0.0096  0.0979      4     no  experiment
##
## Test for Residual Heterogeneity:
## QE(df = 6) = 3.2404, p-val = 0.7781
##
## Test of Moderators (coefficient 2):
## QM(df = 1) = 0.3068, p-val = 0.5796
##
## Model Results:
##
##                 estimate      se     zval    pval    ci.lb   ci.ub
## intrcpt           1.3009  0.2188   5.9469  <.0001   0.8722  1.7296  ***
## contextOnline    -0.1484  0.2680  -0.5539  0.5796  -0.6736  0.3768
##
## ---
## Signif. codes:  0 '***' 0.001 '**' 0.01 '*' 0.05 '.' 0.1 ' ' 1
```

Forest plot.

```
lt = c("Study 1 - In-Person", "Study 1 - Online", "Study 2 - In-Person", "Study 2 - Online", "Study 3 -
ma_mod %>%
  forest(slab = lt, col = "green", xlab = "Observed Effect Size (Cohen's d)")
```

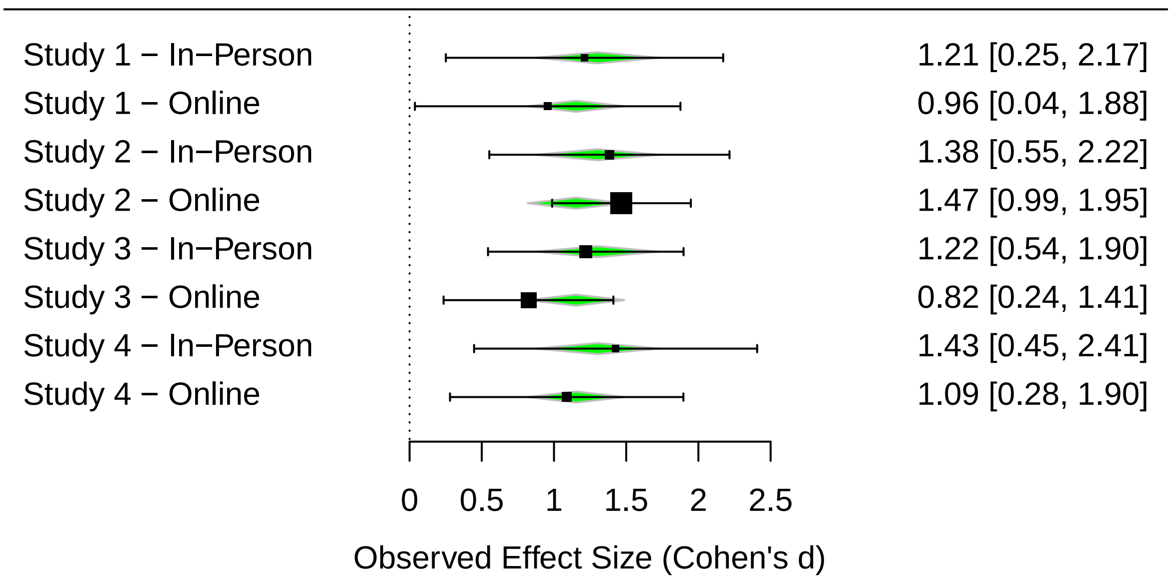

Graphing

Liu Spelke Data Prep

```
liu_graphing <- final.repl.data.avg %>%
  filter(trialtype != "famhab") %>%
  group_by(sample, trialtype) %>%
  summarise(looking_time = mean(avg_look),
            sems = std.error(avg_look))
```

```
## `summarise()` has grouped output by 'sample'. You can override using the `.groups` argument.
```

Woo Spelke Data Prep

```
woo_study1_graphing <- choice.dm %>%
  mutate(Intention = recode(intention,  "Irrelevant\nIntention" = "Irrelevant", "Helpful\nIntention" = 
  mutate(Outcome = recode(outcome, "Unhelpful\nOutcome" = "Unhelpful", "Positive\nOutcome" = "Helpful")
woo_study2 <- dplyr::filter(bh2zoomL) %>%
  mutate(Outcome = recode(Outcome, "Unhelpful\nOutcome" = "Unhelpful", "Positive\nOutcome" = "Helpful")
  mutate(Intention = recode(Intention, "Irrelevant\nIntention" = "Irrelevant", "Helpful\nIntention" = "
woo_study2
```

```
##    Relabeled.Script Script Subject.ID Sex Age...Month Age...Day Age.Decimal
## 1                1b      9        p25   F          15         9    15.30000
## 2                2b     10        p17   F          15         0    15.00000
## 3                3b      5        p26   F          14        10    14.33333
## 4                4b     14        p13   F          15        11    15.36667
## 5                5b      3        p22   M          14        18    14.60000
## 6                6b     12        p15   M          14        25    14.83333
## 7                7b     16        p19   M          14        28    14.93333
## 8                8b      7        p10   F          15         3    15.10000
## 9                9b      1         p9   F          15         9    15.30000
## 10              10b      2        p12   F          15        17    15.56667
## 11              11b     11        p23   M          14        24    14.80000
## 12              12b      4        p14   M          14        27    14.90000
## 13              13b      6        p18   F          14        24    14.80000
## 14              14b      8        p21   F          14        18    14.60000
## 15              15b     13        p24   F          14        20    14.66667
## 16              16b     15      p19.2   M          14        21    14.70000
## 17               1c      7        p43   M          14        12    14.40000
## 18               2c      9        p27   M          14        19    14.63333
## 19               3c      8        p32   F          15        19    15.63333
## 20               4c      2        p35   F          14        27    14.90000
## 21               5c      3        p29   F          14        18    14.60000
## 22               6c      1        p36   M          15        13    15.43333
## 23               7c     13        p34   M          15         4    15.13333
## 24               8c     11        p28   F          14        19    14.63333
## 25               9c     10        p44   M          15        20    15.66667
## 26              10c      4        p37   M          14        14    14.46667
## 27              11c     12        p42   F          14        21    14.70000
## 28              12c     14        p31   M          15         0    15.00000
## 29              13c      6        p41   F          14        19    14.63333
## 30              14c      5        p30   F          14        28    14.93333
## 31              15c     16        p33   F          15        13    15.43333
## 32              16c     15        p39   F          14        11    14.36667
## 33               1d     14        p62   F          14        11    14.36667
## 34               2d     15        p50   M          14        23    14.76667
## 35               3d      1        p56   F          15         1    15.03333
## 36               4d      5        p58   M          14        19    14.63333
## 37               5d      2        p49   F          14        29    14.96667
## 38               6d     16        p40   M          14        21    14.70000
## 39               7d      4        p51   F          14        29    14.96667
```

```
## 40           8d   10     p48  F      14     27  14.90000
## 41           9d   12     p45  M      14     15  14.50000
## 42          10d   11     p52  M      15      7  15.23333
## 43          11d   13     p59  M      14     15  14.50000
## 44          12d    3     p57  M      15      2  15.06667
## 45          13d    9     p53  F      15      2  15.06667
## 46          14d    8     p47  F      14     10  14.33333
## 47          16d    6     p61  M      15     20  15.66667
## 48          15d    3     p55  M      15     13  15.43333
## 49           1b    9     p25  F      15      9  15.30000
## 50           2b   10     p17  F      15      0  15.00000
## 51           3b    5     p26  F      14     10  14.33333
## 52           4b   14     p13  F      15     11  15.36667
## 53           5b    3     p22  M      14     18  14.60000
## 54           6b   12     p15  M      14     25  14.83333
## 55           7b   16     p19  M      14     28  14.93333
## 56           8b    7     p10  F      15      3  15.10000
## 57           9b    1      p9  F      15      9  15.30000
## 58          10b    2     p12  F      15     17  15.56667
## 59          11b   11     p23  M      14     24  14.80000
## 60          12b    4     p14  M      14     27  14.90000
## 61          13b    6     p18  F      14     24  14.80000
## 62          14b    8     p21  F      14     18  14.60000
## 63          15b   13     p24  F      14     20  14.66667
## 64          16b   15   p19.2  M      14     21  14.70000
## 65           1c    7     p43  M      14     12  14.40000
## 66           2c    9     p27  M      14     19  14.63333
## 67           3c    8     p32  F      15     19  15.63333
## 68           4c    2     p35  F      14     27  14.90000
## 69           5c    3     p29  F      14     18  14.60000
## 70           6c    1     p36  M      15     13  15.43333
## 71           7c   13     p34  M      15      4  15.13333
## 72           8c   11     p28  F      14     19  14.63333
## 73           9c   10     p44  M      15     20  15.66667
## 74          10c    4     p37  M      14     14  14.46667
## 75          11c   12     p42  F      14     21  14.70000
## 76          12c   14     p31  M      15      0  15.00000
## 77          13c    6     p41  F      14     19  14.63333
## 78          14c    5     p30  F      14     28  14.93333
## 79          15c   16     p33  F      15     13  15.43333
## 80          16c   15     p39  F      14     11  14.36667
## 81           1d   14     p62  F      14     11  14.36667
## 82           2d   15     p50  M      14     23  14.76667
## 83           3d    1     p56  F      15      1  15.03333
## 84           4d    5     p58  M      14     19  14.63333
## 85           5d    2     p49  F      14     29  14.96667
## 86           6d   16     p40  M      14     21  14.70000
## 87           7d    4     p51  F      14     29  14.96667
## 88           8d   10     p48  F      14     27  14.90000
## 89           9d   12     p45  M      14     15  14.50000
## 90          10d   11     p52  M      15      7  15.23333
## 91          11d   13     p59  M      14     15  14.50000
## 92          12d    3     p57  M      15      2  15.06667
## 93          13d    9     p53  F      15      2  15.06667
```

```
## 94              14d      8        p47   F          14         10    14.33333
## 95              16d      6        p61   M          15         20    15.66667
## 96              15d      3        p55   M          15         13    15.43333
##       Condition Fam.Box.Color Pink.Actor.Show.Side Pink.Actor.Order
## 1  False Belief          Blue                 Left                1
## 2  False Belief          Blue                 Left                1
## 3   True Belief         Green                 Left                2
## 4  False Belief         Green                 Left                2
## 5   True Belief          Blue                Right                2
## 6  False Belief          Blue                Right                2
## 7  False Belief         Green                Right                1
## 8   True Belief         Green                Right                1
## 9   True Belief          Blue                 Left                1
## 10  True Belief          Blue                 Left                1
## 11 False Belief          Blue                Right                2
## 12  True Belief          Blue                Right                2
## 13  True Belief         Green                 Left                2
## 14  True Belief         Green                Right                1
## 15 False Belief         Green                 Left                2
## 16 False Belief         Green                Right                1
## 17  True Belief         Green                Right                1
## 18 False Belief          Blue                 Left                1
## 19  True Belief         Green                Right                1
## 20  True Belief          Blue                 Left                1
## 21  True Belief          Blue                Right                2
## 22  True Belief          Blue                 Left                1
## 23 False Belief         Green                 Left                2
## 24 False Belief          Blue                Right                2
## 25 False Belief          Blue                 Left                1
## 26  True Belief          Blue                Right                2
## 27 False Belief          Blue                Right                2
## 28 False Belief         Green                 Left                2
## 29  True Belief         Green                 Left                2
## 30  True Belief         Green                 Left                2
## 31 False Belief         Green                Right                1
## 32 False Belief         Green                Right                1
## 33 False Belief         Green                 Left                2
## 34 False Belief         Green                Right                1
## 35  True Belief          Blue                 Left                1
## 36  True Belief         Green                 Left                2
## 37  True Belief          Blue                 Left                1
## 38 False Belief         Green                Right                1
## 39  True Belief          Blue                Right                2
## 40 False Belief          Blue                 Left                1
## 41 False Belief          Blue                Right                2
## 42 False Belief          Blue                Right                2
## 43 False Belief         Green                 Left                2
## 44  True Belief          Blue                Right                2
## 45 False Belief          Blue                 Left                1
## 46  True Belief         Green                Right                1
## 47  True Belief         Green                 Left                2
## 48  True Belief         Green                Right                1
## 49 False Belief          Blue                 Left                1
## 50 False Belief          Blue                 Left                1
```

```
## 51  True Belief         Green                 Left                2
## 52 False Belief         Green                 Left                2
## 53  True Belief          Blue                Right                2
## 54 False Belief          Blue                Right                2
## 55 False Belief         Green                Right                1
## 56  True Belief         Green                Right                1
## 57  True Belief          Blue                 Left                1
## 58  True Belief          Blue                 Left                1
## 59 False Belief          Blue                Right                2
## 60  True Belief          Blue                Right                2
## 61  True Belief         Green                 Left                2
## 62  True Belief         Green                Right                1
## 63 False Belief         Green                 Left                2
## 64 False Belief         Green                Right                1
## 65  True Belief         Green                Right                1
## 66 False Belief          Blue                 Left                1
## 67  True Belief         Green                Right                1
## 68  True Belief          Blue                 Left                1
## 69  True Belief          Blue                Right                2
## 70  True Belief          Blue                 Left                1
## 71 False Belief         Green                 Left                2
## 72 False Belief          Blue                Right                2
## 73 False Belief          Blue                 Left                1
## 74  True Belief          Blue                Right                2
## 75 False Belief          Blue                Right                2
## 76 False Belief         Green                 Left                2
## 77  True Belief         Green                 Left                2
## 78  True Belief         Green                 Left                2
## 79 False Belief         Green                Right                1
## 80 False Belief         Green                Right                1
## 81 False Belief         Green                 Left                2
## 82 False Belief         Green                Right                1
## 83  True Belief          Blue                 Left                1
## 84  True Belief         Green                 Left                2
## 85  True Belief          Blue                 Left                1
## 86 False Belief         Green                Right                1
## 87  True Belief          Blue                Right                2
## 88 False Belief          Blue                 Left                1
## 89 False Belief          Blue                Right                2
## 90 False Belief          Blue                Right                2
## 91 False Belief         Green                 Left                2
## 92  True Belief          Blue                Right                2
## 93 False Belief          Blue                 Left                1
## 94  True Belief         Green                Right                1
## 95  True Belief         Green                 Left                2
## 96  True Belief         Green                Right                1
##    Test.Actor.Old.Toy.Color Pink.Actor.Choice.Side ChoiceLeft ChoiceRight
## 1                      Pink                   Left      15.29       12.49
## 2                    Yellow                   Left       7.60       18.51
## 3                    Yellow                   Left       9.87       17.94
## 4                      Pink                   Left      10.61       11.84
## 5                      Pink                  Right      11.66       12.55
## 6                    Yellow                  Right      10.03       16.96
## 7                      Pink                  Right      14.25        8.94
```

```
## 8                         Yellow                   Right       7.86        4.72
## 9                           Pink                    Left      18.44        7.19
## 10                        Yellow                    Left       3.30       22.50
## 11                          Pink                   Right      15.59       12.48
## 12                        Yellow                   Right       9.81       16.42
## 13                          Pink                    Left      11.42       15.85
## 14                          Pink                   Right       8.62       17.75
## 15                        Yellow                    Left      14.85       11.47
## 16                        Yellow                   Right       7.90       16.05
## 17                        Yellow                   Right      13.06        7.73
## 18                          Pink                    Left       8.25       14.36
## 19                          Pink                   Right       4.76       22.48
## 20                        Yellow                    Left       9.77       14.34
## 21                          Pink                   Right       8.13       11.85
## 22                          Pink                    Left       9.59       18.24
## 23                        Yellow                    Left      18.32        9.42
## 24                          Pink                   Right      18.45       10.09
## 25                        Yellow                    Left      12.38        2.65
## 26                        Yellow                   Right      16.15       11.59
## 27                        Yellow                   Right       8.07        9.44
## 28                          Pink                    Left       7.53       17.10
## 29                          Pink                    Left      11.53       14.74
## 30                        Yellow                    Left       8.76       18.47
## 31                          Pink                   Right      15.42       11.20
## 32                        Yellow                   Right      10.71       14.66
## 33                          Pink                    Left       6.50       14.59
## 34                        Yellow                   Right       8.16       12.12
## 35                          Pink                    Left       4.07        9.05
## 36                        Yellow                    Left       5.72       14.37
## 37                        Yellow                    Left       5.92       12.45
## 38                          Pink                   Right      11.48       15.16
## 39                        Yellow                   Right       6.58       13.52
## 40                        Yellow                    Left      17.84        6.55
## 41                        Yellow                   Right       6.58        5.44
## 42                          Pink                   Right       6.11       23.68
## 43                        Yellow                    Left       8.26       14.07
## 44                          Pink                   Right      10.01       12.36
## 45                          Pink                    Left       6.11       19.53
## 46                          Pink                   Right       6.70        9.74
## 47                          Pink                    Left       6.62        3.70
## 48                        Yellow                   Right      16.24        9.11
## 49                          Pink                    Left      15.29       12.49
## 50                        Yellow                    Left       7.60       18.51
## 51                        Yellow                    Left       9.87       17.94
## 52                          Pink                    Left      10.61       11.84
## 53                          Pink                   Right      11.66       12.55
## 54                        Yellow                   Right      10.03       16.96
## 55                          Pink                   Right      14.25        8.94
## 56                        Yellow                   Right       7.86        4.72
## 57                          Pink                    Left      18.44        7.19
## 58                        Yellow                    Left       3.30       22.50
## 59                          Pink                   Right      15.59       12.48
## 60                        Yellow                   Right       9.81       16.42
## 61                          Pink                    Left      11.42       15.85
```

```
## 62                     Pink                     Right       8.62       17.75
## 63                   Yellow                      Left      14.85       11.47
## 64                   Yellow                     Right       7.90       16.05
## 65                   Yellow                     Right      13.06        7.73
## 66                     Pink                      Left       8.25       14.36
## 67                     Pink                     Right       4.76       22.48
## 68                   Yellow                      Left       9.77       14.34
## 69                     Pink                     Right       8.13       11.85
## 70                     Pink                      Left       9.59       18.24
## 71                   Yellow                      Left      18.32        9.42
## 72                     Pink                     Right      18.45       10.09
## 73                   Yellow                      Left      12.38        2.65
## 74                   Yellow                     Right      16.15       11.59
## 75                   Yellow                     Right       8.07        9.44
## 76                     Pink                      Left       7.53       17.10
## 77                     Pink                      Left      11.53       14.74
## 78                   Yellow                      Left       8.76       18.47
## 79                     Pink                     Right      15.42       11.20
## 80                   Yellow                     Right      10.71       14.66
## 81                     Pink                      Left       6.50       14.59
## 82                   Yellow                     Right       8.16       12.12
## 83                     Pink                      Left       4.07        9.05
## 84                   Yellow                      Left       5.72       14.37
## 85                   Yellow                      Left       5.92       12.45
## 86                     Pink                     Right      11.48       15.16
## 87                   Yellow                     Right       6.58       13.52
## 88                   Yellow                      Left      17.84        6.55
## 89                   Yellow                     Right       6.58        5.44
## 90                     Pink                     Right       6.11       23.68
## 91                   Yellow                      Left       8.26       14.07
## 92                     Pink                     Right      10.01       12.36
## 93                     Pink                      Left       6.11       19.53
## 94                     Pink                     Right       6.70        9.74
## 95                     Pink                      Left       6.62        3.70
## 96                   Yellow                     Right      16.24        9.11
##    pPositiveO pUnhelpfulO pIrrelevantI pHelpfulI        ActorType    LT
## 1   0.5503960   0.4496040    0.5503960 0.4496040  Positive-Outcome 15.29
## 2   0.7089238   0.2910762    0.7089238 0.2910762  Positive-Outcome 18.51
## 3   0.6450917   0.3549083    0.3549083 0.6450917  Positive-Outcome 17.94
## 4   0.4726058   0.5273942    0.4726058 0.5273942  Positive-Outcome 10.61
## 5   0.5183808   0.4816192    0.4816192 0.5183808  Positive-Outcome 12.55
## 6   0.3716191   0.6283809    0.3716191 0.6283809  Positive-Outcome 10.03
## 7   0.3855110   0.6144890    0.3855110 0.6144890  Positive-Outcome  8.94
## 8   0.6248013   0.3751987    0.3751987 0.6248013  Positive-Outcome  7.86
## 9   0.7194694   0.2805306    0.2805306 0.7194694  Positive-Outcome 18.44
## 10  0.8720930   0.1279070    0.1279070 0.8720930  Positive-Outcome 22.50
## 11  0.4446028   0.5553972    0.4446028 0.5553972  Positive-Outcome 12.48
## 12  0.3739992   0.6260008    0.6260008 0.3739992  Positive-Outcome  9.81
## 13  0.4187752   0.5812248    0.5812248 0.4187752  Positive-Outcome 11.42
## 14  0.6731134   0.3268866    0.3268866 0.6731134  Positive-Outcome 17.75
## 15  0.4357903   0.5642097    0.4357903 0.5642097  Positive-Outcome 11.47
## 16  0.3298539   0.6701461    0.3298539 0.6701461  Positive-Outcome  7.90
## 17  0.6281866   0.3718134    0.3718134 0.6281866  Positive-Outcome 13.06
## 18  0.3648828   0.6351172    0.3648828 0.6351172  Positive-Outcome  8.25
```

```
## 19  0.8252570   0.1747430    0.1747430 0.8252570  Positive-Outcome 22.48
## 20  0.5947740   0.4052260    0.4052260 0.5947740  Positive-Outcome 14.34
## 21  0.5930931   0.4069069    0.4069069 0.5930931  Positive-Outcome 11.85
## 22  0.3445922   0.6554078    0.6554078 0.3445922  Positive-Outcome  9.59
## 23  0.3395818   0.6604182    0.3395818 0.6604182  Positive-Outcome  9.42
## 24  0.3535389   0.6464611    0.3535389 0.6464611  Positive-Outcome 10.09
## 25  0.1763140   0.8236860    0.1763140 0.8236860  Positive-Outcome  2.65
## 26  0.5821918   0.4178082    0.4178082 0.5821918  Positive-Outcome 16.15
## 27  0.4608795   0.5391205    0.4608795 0.5391205  Positive-Outcome  8.07
## 28  0.3057247   0.6942753    0.3057247 0.6942753  Positive-Outcome  7.53
## 29  0.4389037   0.5610963    0.5610963 0.4389037  Positive-Outcome 11.53
## 30  0.6782960   0.3217040    0.3217040 0.6782960  Positive-Outcome 18.47
## 31  0.4207363   0.5792637    0.4207363 0.5792637  Positive-Outcome 11.20
## 32  0.4221521   0.5778479    0.4221521 0.5778479  Positive-Outcome 10.71
## 33  0.3082029   0.6917971    0.3082029 0.6917971  Positive-Outcome  6.50
## 34  0.4023669   0.5976331    0.4023669 0.5976331  Positive-Outcome  8.16
## 35  0.3102134   0.6897866    0.6897866 0.3102134  Positive-Outcome  4.07
## 36  0.7152812   0.2847188    0.2847188 0.7152812  Positive-Outcome 14.37
## 37  0.6777354   0.3222646    0.3222646 0.6777354  Positive-Outcome 12.45
## 38  0.5690691   0.4309309    0.5690691 0.4309309  Positive-Outcome 15.16
## 39  0.3273632   0.6726368    0.6726368 0.3273632  Positive-Outcome  6.58
## 40  0.2685527   0.7314473    0.2685527 0.7314473  Positive-Outcome  6.55
## 41  0.5474210   0.4525790    0.5474210 0.4525790  Positive-Outcome  6.58
## 42  0.7948976   0.2051024    0.7948976 0.2051024  Positive-Outcome 23.68
## 43  0.6300940   0.3699060    0.6300940 0.3699060  Positive-Outcome 14.07
## 44  0.5525257   0.4474743    0.4474743 0.5525257  Positive-Outcome 12.36
## 45  0.2382995   0.7617005    0.2382995 0.7617005  Positive-Outcome  6.11
## 46  0.5924574   0.4075426    0.4075426 0.5924574  Positive-Outcome  9.74
## 47  0.6414729   0.3585271    0.3585271 0.6414729  Positive-Outcome  6.62
## 48  0.6406312   0.3593688    0.3593688 0.6406312  Positive-Outcome 16.24
## 49  0.5503960   0.4496040    0.5503960 0.4496040 Unhelpful-Outcome 12.49
## 50  0.7089238   0.2910762    0.7089238 0.2910762 Unhelpful-Outcome  7.60
## 51  0.6450917   0.3549083    0.3549083 0.6450917 Unhelpful-Outcome  9.87
## 52  0.4726058   0.5273942    0.4726058 0.5273942 Unhelpful-Outcome 11.84
## 53  0.5183808   0.4816192    0.4816192 0.5183808 Unhelpful-Outcome 11.66
## 54  0.3716191   0.6283809    0.3716191 0.6283809 Unhelpful-Outcome 16.96
## 55  0.3855110   0.6144890    0.3855110 0.6144890 Unhelpful-Outcome 14.25
## 56  0.6248013   0.3751987    0.3751987 0.6248013 Unhelpful-Outcome  4.72
## 57  0.7194694   0.2805306    0.2805306 0.7194694 Unhelpful-Outcome  7.19
## 58  0.8720930   0.1279070    0.1279070 0.8720930 Unhelpful-Outcome  3.30
## 59  0.4446028   0.5553972    0.4446028 0.5553972 Unhelpful-Outcome 15.59
## 60  0.3739992   0.6260008    0.6260008 0.3739992 Unhelpful-Outcome 16.42
## 61  0.4187752   0.5812248    0.5812248 0.4187752 Unhelpful-Outcome 15.85
## 62  0.6731134   0.3268866    0.3268866 0.6731134 Unhelpful-Outcome  8.62
## 63  0.4357903   0.5642097    0.4357903 0.5642097 Unhelpful-Outcome 14.85
## 64  0.3298539   0.6701461    0.3298539 0.6701461 Unhelpful-Outcome 16.05
## 65  0.6281866   0.3718134    0.3718134 0.6281866 Unhelpful-Outcome  7.73
## 66  0.3648828   0.6351172    0.3648828 0.6351172 Unhelpful-Outcome 14.36
## 67  0.8252570   0.1747430    0.1747430 0.8252570 Unhelpful-Outcome  4.76
## 68  0.5947740   0.4052260    0.4052260 0.5947740 Unhelpful-Outcome  9.77
## 69  0.5930931   0.4069069    0.4069069 0.5930931 Unhelpful-Outcome  8.13
## 70  0.3445922   0.6554078    0.6554078 0.3445922 Unhelpful-Outcome 18.24
## 71  0.3395818   0.6604182    0.3395818 0.6604182 Unhelpful-Outcome 18.32
## 72  0.3535389   0.6464611    0.3535389 0.6464611 Unhelpful-Outcome 18.45
```

```
## 73  0.1763140   0.8236860    0.1763140 0.8236860 Unhelpful-Outcome 12.38
## 74  0.5821918   0.4178082    0.4178082 0.5821918 Unhelpful-Outcome 11.59
## 75  0.4608795   0.5391205    0.4608795 0.5391205 Unhelpful-Outcome  9.44
## 76  0.3057247   0.6942753    0.3057247 0.6942753 Unhelpful-Outcome 17.10
## 77  0.4389037   0.5610963    0.5610963 0.4389037 Unhelpful-Outcome 14.74
## 78  0.6782960   0.3217040    0.3217040 0.6782960 Unhelpful-Outcome  8.76
## 79  0.4207363   0.5792637    0.4207363 0.5792637 Unhelpful-Outcome 15.42
## 80  0.4221521   0.5778479    0.4221521 0.5778479 Unhelpful-Outcome 14.66
## 81  0.3082029   0.6917971    0.3082029 0.6917971 Unhelpful-Outcome 14.59
## 82  0.4023669   0.5976331    0.4023669 0.5976331 Unhelpful-Outcome 12.12
## 83  0.3102134   0.6897866    0.6897866 0.3102134 Unhelpful-Outcome  9.05
## 84  0.7152812   0.2847188    0.2847188 0.7152812 Unhelpful-Outcome  5.72
## 85  0.6777354   0.3222646    0.3222646 0.6777354 Unhelpful-Outcome  5.92
## 86  0.5690691   0.4309309    0.5690691 0.4309309 Unhelpful-Outcome 11.48
## 87  0.3273632   0.6726368    0.6726368 0.3273632 Unhelpful-Outcome 13.52
## 88  0.2685527   0.7314473    0.2685527 0.7314473 Unhelpful-Outcome 17.84
## 89  0.5474210   0.4525790    0.5474210 0.4525790 Unhelpful-Outcome  5.44
## 90  0.7948976   0.2051024    0.7948976 0.2051024 Unhelpful-Outcome  6.11
## 91  0.6300940   0.3699060    0.6300940 0.3699060 Unhelpful-Outcome  8.26
## 92  0.5525257   0.4474743    0.4474743 0.5525257 Unhelpful-Outcome 10.01
## 93  0.2382995   0.7617005    0.2382995 0.7617005 Unhelpful-Outcome 19.53
## 94  0.5924574   0.4075426    0.4075426 0.5924574 Unhelpful-Outcome  6.70
## 95  0.6414729   0.3585271    0.3585271 0.6414729 Unhelpful-Outcome  3.70
## 96  0.6406312   0.3593688    0.3593688 0.6406312 Unhelpful-Outcome  9.11
##      Outcome  Intention
## 1    Helpful Irrelevant
## 2    Helpful Irrelevant
## 3    Helpful    Helpful
## 4    Helpful Irrelevant
## 5    Helpful    Helpful
## 6    Helpful Irrelevant
## 7    Helpful Irrelevant
## 8    Helpful    Helpful
## 9    Helpful    Helpful
## 10   Helpful    Helpful
## 11   Helpful Irrelevant
## 12   Helpful    Helpful
## 13   Helpful    Helpful
## 14   Helpful    Helpful
## 15   Helpful Irrelevant
## 16   Helpful Irrelevant
## 17   Helpful    Helpful
## 18   Helpful Irrelevant
## 19   Helpful    Helpful
## 20   Helpful    Helpful
## 21   Helpful    Helpful
## 22   Helpful    Helpful
## 23   Helpful Irrelevant
## 24   Helpful Irrelevant
## 25   Helpful Irrelevant
## 26   Helpful    Helpful
## 27   Helpful Irrelevant
## 28   Helpful Irrelevant
## 29   Helpful    Helpful
```

```
## 30   Helpful    Helpful
## 31   Helpful Irrelevant
## 32   Helpful Irrelevant
## 33   Helpful Irrelevant
## 34   Helpful Irrelevant
## 35   Helpful    Helpful
## 36   Helpful    Helpful
## 37   Helpful    Helpful
## 38   Helpful Irrelevant
## 39   Helpful    Helpful
## 40   Helpful Irrelevant
## 41   Helpful Irrelevant
## 42   Helpful Irrelevant
## 43   Helpful Irrelevant
## 44   Helpful    Helpful
## 45   Helpful Irrelevant
## 46   Helpful    Helpful
## 47   Helpful    Helpful
## 48   Helpful    Helpful
## 49 Unhelpful    Helpful
## 50 Unhelpful    Helpful
## 51 Unhelpful Irrelevant
## 52 Unhelpful    Helpful
## 53 Unhelpful Irrelevant
## 54 Unhelpful    Helpful
## 55 Unhelpful    Helpful
## 56 Unhelpful Irrelevant
## 57 Unhelpful Irrelevant
## 58 Unhelpful Irrelevant
## 59 Unhelpful    Helpful
## 60 Unhelpful Irrelevant
## 61 Unhelpful Irrelevant
## 62 Unhelpful Irrelevant
## 63 Unhelpful    Helpful
## 64 Unhelpful    Helpful
## 65 Unhelpful Irrelevant
## 66 Unhelpful    Helpful
## 67 Unhelpful Irrelevant
## 68 Unhelpful Irrelevant
## 69 Unhelpful Irrelevant
## 70 Unhelpful Irrelevant
## 71 Unhelpful    Helpful
## 72 Unhelpful    Helpful
## 73 Unhelpful    Helpful
## 74 Unhelpful Irrelevant
## 75 Unhelpful    Helpful
## 76 Unhelpful    Helpful
## 77 Unhelpful Irrelevant
## 78 Unhelpful Irrelevant
## 79 Unhelpful    Helpful
## 80 Unhelpful    Helpful
## 81 Unhelpful    Helpful
## 82 Unhelpful    Helpful
## 83 Unhelpful Irrelevant
```

```
## 84 Unhelpful Irrelevant
## 85 Unhelpful Irrelevant
## 86 Unhelpful    Helpful
## 87 Unhelpful Irrelevant
## 88 Unhelpful    Helpful
## 89 Unhelpful    Helpful
## 90 Unhelpful    Helpful
## 91 Unhelpful    Helpful
## 92 Unhelpful Irrelevant
## 93 Unhelpful    Helpful
## 94 Unhelpful Irrelevant
## 95 Unhelpful Irrelevant
## 96 Unhelpful Irrelevant
```

```
woo_study2_graphing <- woo_study2 %>%
  group_by(Condition, Outcome) %>%
  summarise(looking_time = mean(LT),
            sems = std.error(LT))
```

```
## `summarise()` has grouped output by 'Condition'. You can override using the `.groups` argument.
```

Leonard Data Prep

```
leonard_graphing = leonard_data %>%
  group_by(version) %>%
  summarise(correct = mean(test_question),
            sems = std.error(test_question))
```

Asaba Wu Data Prep

```
asaba_graphing = asaba_data %>%
  group_by(location, outcome) %>%
  summarise(surprise_choice = mean(chose_surprise),
            sems = std.error(chose_surprise))
```

```
## `summarise()` has grouped output by 'location'. You can override using the `.groups` argument.
```

Graphing

```
#liu spelke graph
ls1 <- ggplot(data = liu_graphing, aes(x = sample, y = looking_time, fill = trialtype)) +
  geom_bar(position="dodge", stat="identity") +
  geom_errorbar(aes(ymin = (looking_time - sems), ymax = (looking_time + sems)), position = position_do
  xlab("Data Collection Method") +
  ylab("Looking Time") +
  scale_fill_grey(start = .3, end = .8, name = "Outcome") +
```

```
  theme_minimal()  +
  ggtitle("Study 4")

#woo spelke graph - inperson
w1 <- ggplot(woo_study1_graphing, aes(x = Condition, y = value, fill = Outcome)) +
  geom_bar(position="dodge", stat="identity") +
  labs(x = "Condition", y = "Number of Infants Reaching") +
  theme_minimal() +
  ggtitle("Study 3 - In-Person") +
  scale_fill_grey(start = .3, end = .8, labels = c("Unhelpful", "Helpful")) +
  ylim(c(0,25))

#woo spelke graph - online
w2 <- ggplot(woo_study2_graphing,
                          aes(x = Condition, y = looking_time, fill = Outcome)) +
  geom_bar(position="dodge", stat="identity") +
  geom_errorbar(aes(ymin = (looking_time - sems), ymax = (looking_time + sems)), position = position_do
  xlab("Condition") +
  ylab("Looking Time") +
  theme_minimal() +
  ggtitle("Study 3 - Online") +
  scale_fill_grey(start = .3, end = .8, name = "Outcome")

#leonard graph
l1 <- ggplot(data = leonard_graphing, aes(x = version, y = correct)) +
  geom_bar(position="dodge", stat="identity", fill = "grey80") +
  geom_errorbar(aes(ymin = (correct - sems), ymax = (correct + sems)), position = position_dodge(.9), w
  xlab("Data Collection Method") +
  ylab("Prop Correct") +
  geom_hline(linetype = "dashed", yintercept = 0.5) + ylim(0,1) +
  theme_minimal()  +
  ggtitle("Study 1")

#asaba wu grpah
a1 <- ggplot(data = asaba_graphing, aes(x = location, y = surprise_choice, fill = outcome)) +
  geom_bar(position="dodge", stat="identity") +
  geom_errorbar(aes(ymin = (surprise_choice - sems), ymax = (surprise_choice + sems)), position = posit
  xlab("Data Collection Method") +
  ylab("Prop Surprise Choice") +
  geom_hline(linetype = "dashed", yintercept = 0.5) + ylim(0,1) +
  theme_minimal()  +
  ggtitle("Study 2") +
  scale_fill_grey(start = .3, end = .8, name = "Outcome")

#cowplot::plot_grid(w1, w2, ls1, l1, a1)
ggarrange(l1, a1, arrangeGrob(w1, w2, ncol = 2), ls1,
          labels = c("1", "2", "3", "4"),
          ncol = 2, nrow = 2)
```

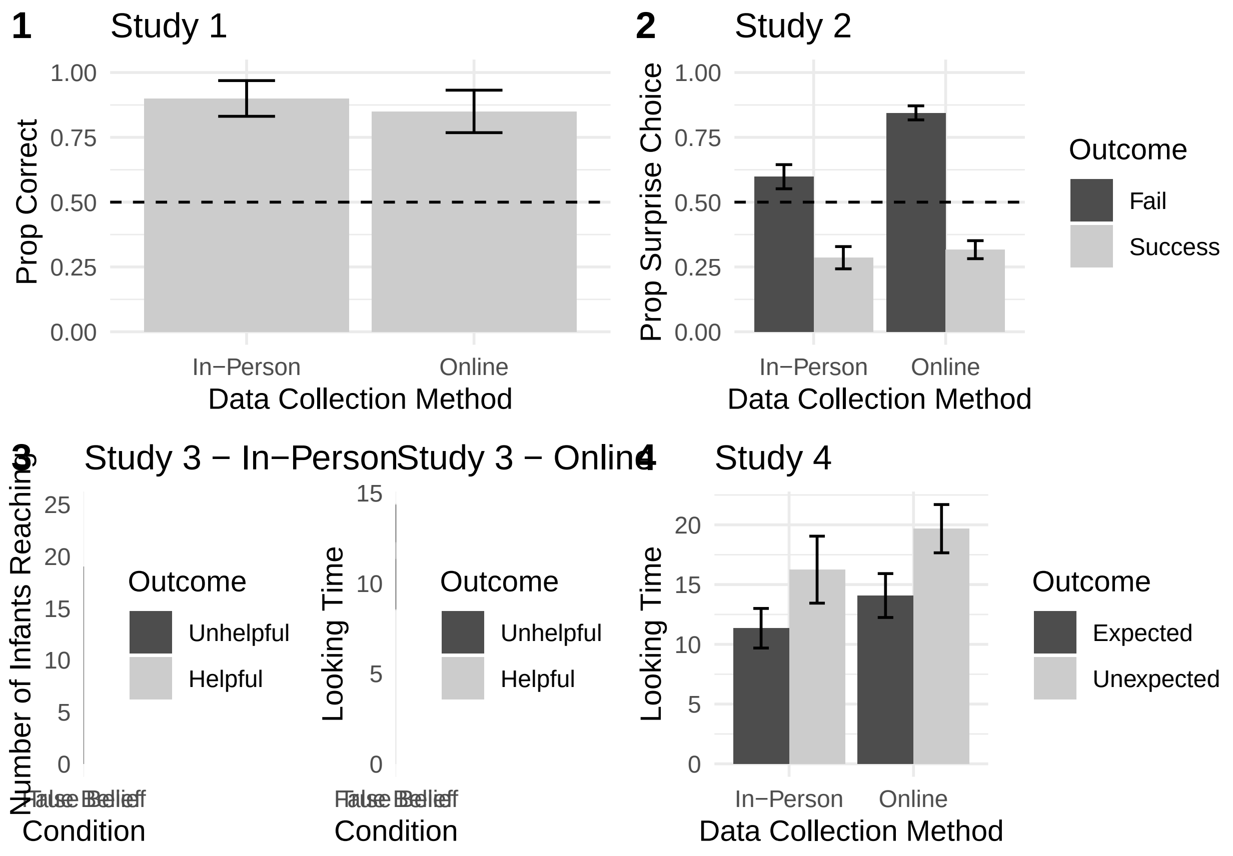
1
Study 1
Prop Correct
1.00
0.75
0.50
0.25
0.00
In−Person
Online
Data Collection Method
2
Study 2
Prop Surprise Choice
1.00
0.75
0.50
0.25
0.00
In−Person
Online
Data Collection Method
Outcome
Fail
Success
3
Study 3 − In−Person
Number of Infants Reaching
25
20
15
10
5
0
False Belief
True Belief
Condition
Outcome
Unhelpful
Helpful
Study 3 − Online
Looking Time
15
10
5
0
False Belief
True Belief
Condition
Outcome
Unhelpful
Helpful
4
Study 4
Looking Time
20
15
10
5
0
In−Person
Online
Data Collection Method
Outcome
Expected
Unexpected
